# Supplementary material for: Unveiling P. vivax invasion pathways in Duffy-negative individuals
Source: Cell Host Microbe. 2023 Dec 13;31(12):2080–2092.e5. doi: 10.1016/j.chom.2023.11.007 (PMC10727064; doi:10.1016/j.chom.2023.11.007)
Supplement: Document S1. Figures S1–S7 and Table S1 [file mmc1.pdf]

Cell Host & Microbe, Volume *31*

Supplemental information

Unveiling *P. vivax* invasion pathways in Duffy-negative individuals

Isabelle Bouyssou, Sara El Hoss, Cécile Doderer-Lang, Matthieu Schoenhals, Lova Tsikiniaina Rasoloharimanana, Inès Vigan-Womas, Arsène Ratsimbasoa, Andargie Abate, Lemu Golassa, Solenne Mabilotte, Pascal Kessler, Micheline Guillotte-Blisnick, Francisco J. Martinez, Chetan E. Chitnis, John Strouboulis, and Didier Ménard

Supplemental information

Unveiling *P. vivax* invasion pathways in Duffy-negative individuals

Isabelle Bouyssou, Sara El Hoss, Cécile Doderer-Lang, Matthieu Schoenhals, Lova Tsikiniaina Rasoloharimanana, Inès Vigan-Womas, Arsène Ratsimbasoa, Andargie Abate, Lemu Golassa, Solenne Mabilotte, Pascal Kessler, Micheline Guillotte-Blisnick, Francisco J. Martinez, Chetan E. Chitnis, John Strouboulis and Didier Ménard

Supplemental materials

Table S1 (Related to Figure 4): List of *P. vivax* isolates and erythroblasts used for *in vitro* invasion assays.

***P. vivax* isolate**	**Origin**	**Invasion Assay**	**HSC source**	***P. vivax* infection detection**					**Shown in**	**Comment**
				**MGG staining**	**18S rRNA FISH probe**	**anti-HSP70**	**18S rRNA FISH probe & anti-HSP70**	**Successful**		
AW007	Ethiopia	No. 6	DP010	Negative	**Positive**	Negative	Not done	**Yes**		
			DN005	Negative	Negative	Negative	Not done	No		
MC4023	Ethiopia	No. 5	DP007	**Positive**	**Positive**	Negative	Not done	**Yes**	Figure S7B	Tiny pellet obtained after thawing of the cryoisolate
			DN006	Negative	Negative	Negative	Not done	No		
MC4038	Ethiopia	No. 6	DP010	**Positive**	**Positive**	**Positive**	**Positive**	**Yes**	Figure 4B	
			DN005	**Positive**	**Positive**	**Positive**	**Positive**	**Yes**	Figure 4C	
MC4043	Ethiopia	No. 5	DP007	Negative	Negative	Negative	Not done	No		Lots of red blood cell aggregates
			DN006	Negative	Negative	Negative	Not done	No		
MC4049	Ethiopia	No. 5	DP007	**Positive**	Negative	Negative	Not done	**Yes**		
			DN006	Negative	Negative	Negative	Not done	No		
WN5042	Ethiopia	No. 6	DP010	**Positive**	**Positive**	Negative	Negative	**Yes**	Figure S6C	
			DN005	**Positive**	**Positive**	Negative	Negative	**Yes**	Figure S7A	
WN5050	Ethiopia	No. 5	DP007	Negative	Negative	Negative	Not done	No		Lots of red blood cell aggregates
			DN006	**Positive**	Negative	**Positive**	Not done	**Yes**	Figure S7C	
MDZV02004	Madagascar	No. 5	DP007	Negative	Not done	Not done	Not done	No		Tiny pellet obtained after thawing of the cryoisolate
			DN006	Negative	Negative	Negative	Not done	No		
		No. 6	DP010	Negative	Negative	**Positive**	Not done	**Yes**	Figure S6A	
			DN005	Negative	Negative	**Positive**	Not done	**Yes**	Figure S6B	
MDZV02005	Madagascar	No. 5	DP007	**Positive**	**Positive**	**Positive**	**Positive**	**Yes**	Figure 4E	
			DN006	**Positive**	Negative	**Positive**	Negative	**Yes**	Figure 4G	
		No. 6	DP010	**Positive**	**Positive**	Negative	Negative	**Yes**	Figure 4D	
			DN005	**Positive**	**Positive**	**Positive**	**Positive**	**Yes**	Figure 4F	

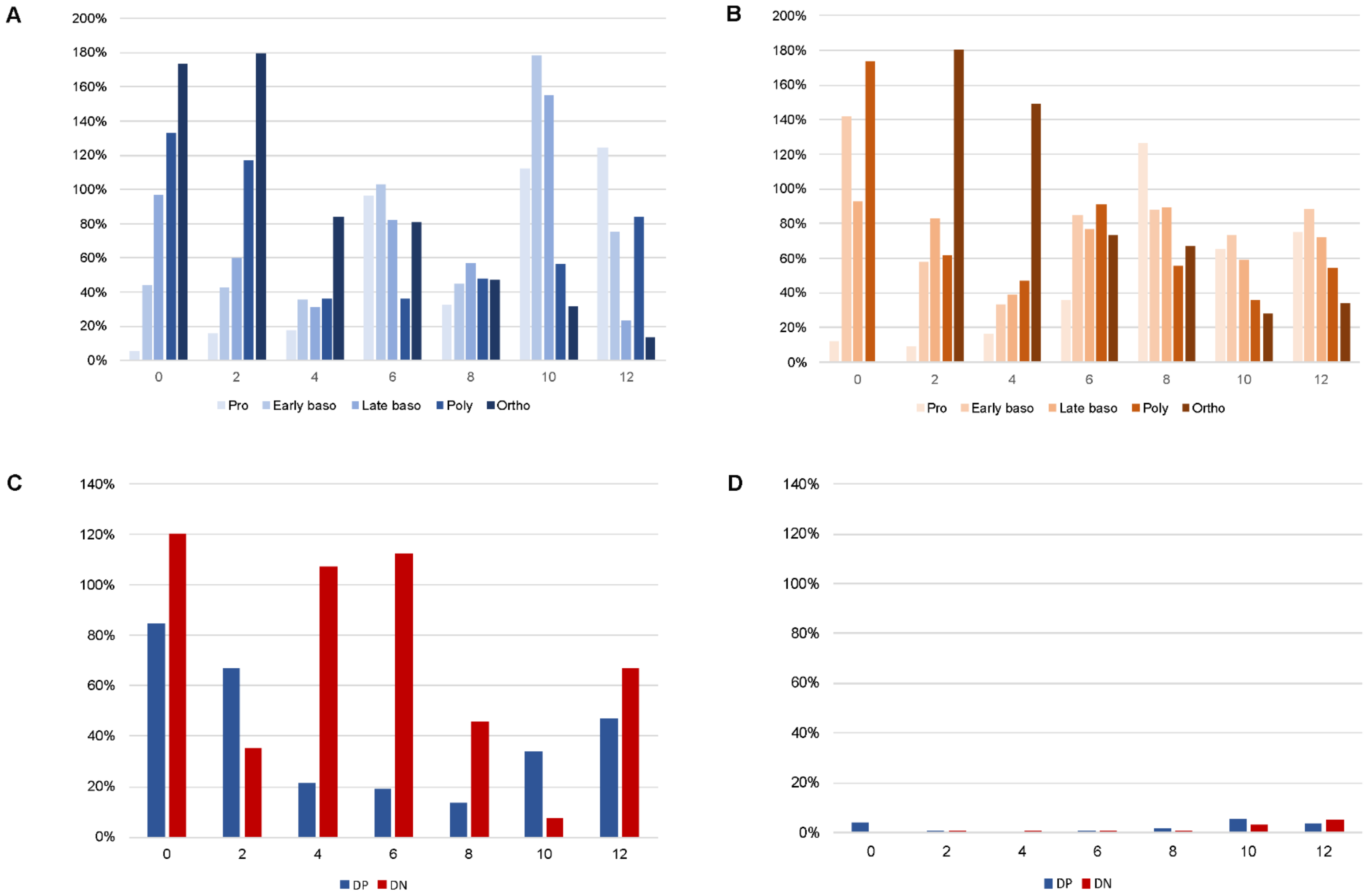
A
200%
180%
160%
140%
120%
100%
80%
60%
40%
20%
0%
0
2
4
6
8
10
12
Pro
Early baso
Late baso
Poly
Ortho
B
200%
180%
160%
140%
120%
100%
80%
60%
40%
20%
0%
0
2
4
6
8
10
12
Pro
Early baso
Late baso
Poly
Ortho
C
140%
120%
100%
80%
60%
40%
20%
0%
0
2
4
6
8
10
12
DP
DN
D
140%
120%
100%
80%
60%
40%
20%
0%
0
2
4
6
8
10
12
DP
DN

Figure S1 (Related to Figure 1 and Figure 2): Inter-individual variation within Duffy-positive (DP) and Duffy-negative (DN) erythroblasts was expressed as relative standard deviations [RSD, RSD=100 × (SD/mean)]. A. RSD of the proportions of the DP erythroblast stages from D0 to D12. **B.** RSD of the proportions of the DN erythroblast stages from D0 to D12. **C.** RSD of the proportion of DP and DN DARC+ erythroblast stages from D0 to D12. **D.** RSD of the proportion of the DP and DN CD71+ erythroblast stages from D0 to D12.

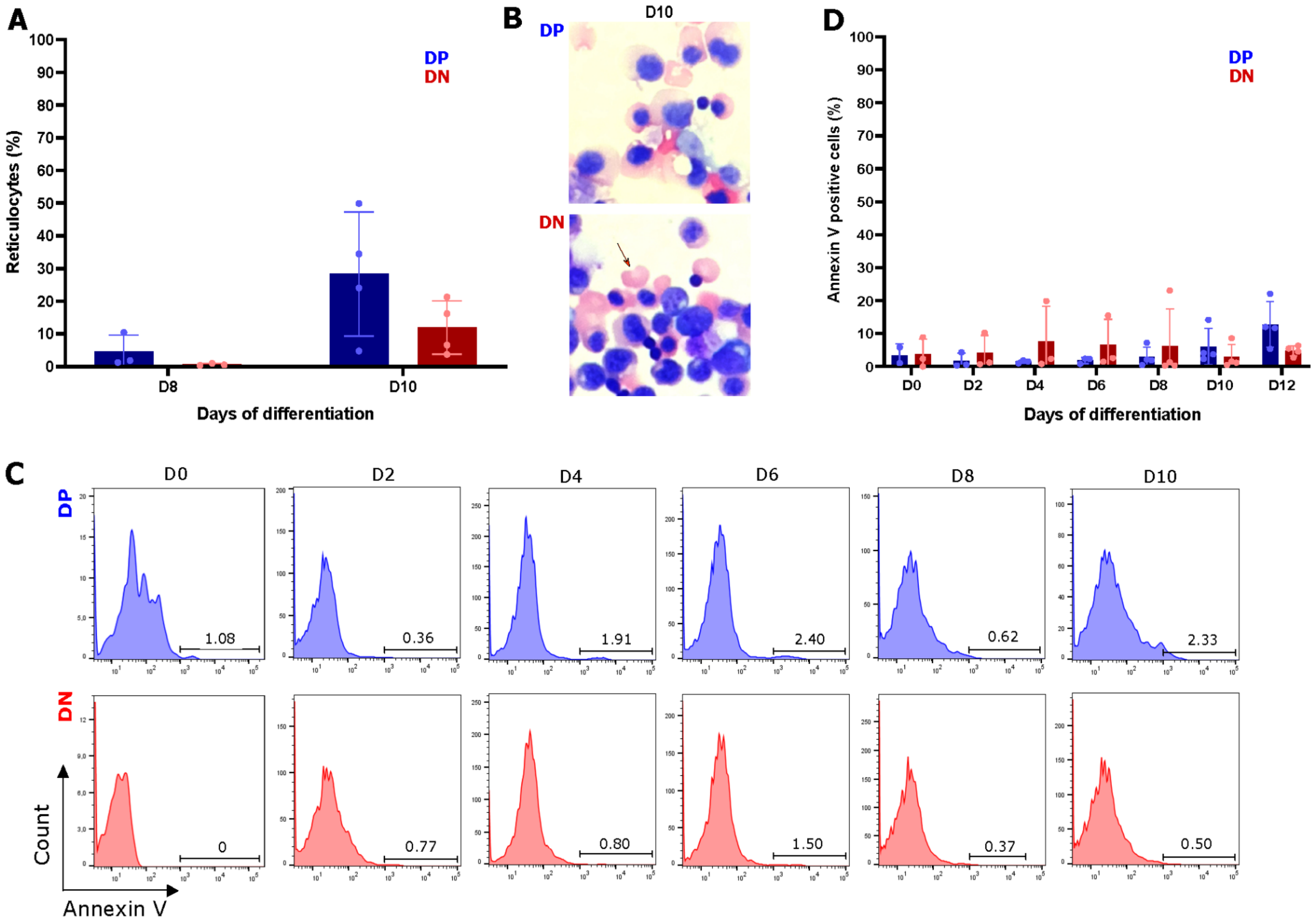
A
Reticulocytes (%)
DP
DN
D8
D10
Days of differentiation
B
D10
DP
DN
D
Annexin V positive cells (%)
DP
DN
D0
D2
D4
D6
D8
D10
D12
Days of differentiation
C
D0
D2
D4
D6
D8
D10
DP
DN
1.08
0.36
1.91
2.40
0.62
2.33
0
0.77
0.80
1.50
0.37
0.50
Count
Annexin V

Figure S2 (Related to Figure 1): Enucleation and apoptosis of Duffy-positive (DP) and Duffy-negative (DN) erythroblasts during terminal differentiation *in vitro*. **A.** The bar chart represents the percentage of reticulocytes in four DP (*DP001, DP002, DP003, DP004*) and four DN (*DN001, DN002, DN003, DN005*) donors at D8 and D10 (mean ±SD) (p=0.2, Mann-Whitney test). **B.** May-Grünwald Giemsa staining showing one DP (*DP001*) and one DN (*DN001*) donors at D10 of terminal differentiation. Arrow indicates the presence of reticulocytes. **C.** Representative histograms showing the percentage of GPA+ Annexin + cells (apoptotic cells) in one DP (*DP003*) and one DN (*DN003*) donor at D0, D2, D4, D6, D8, D10, and D12. **D.** The bar graph represents the percentage of GPA+ Annexin + cells (apoptotic cells) in four DP (*DP001, DP002, DP003, DP004*) and four DN (*DN001, DN002, DN003, DN005*) donors at D0, D2, D4, D6, D8, D10 and D12 (mean ±SD).

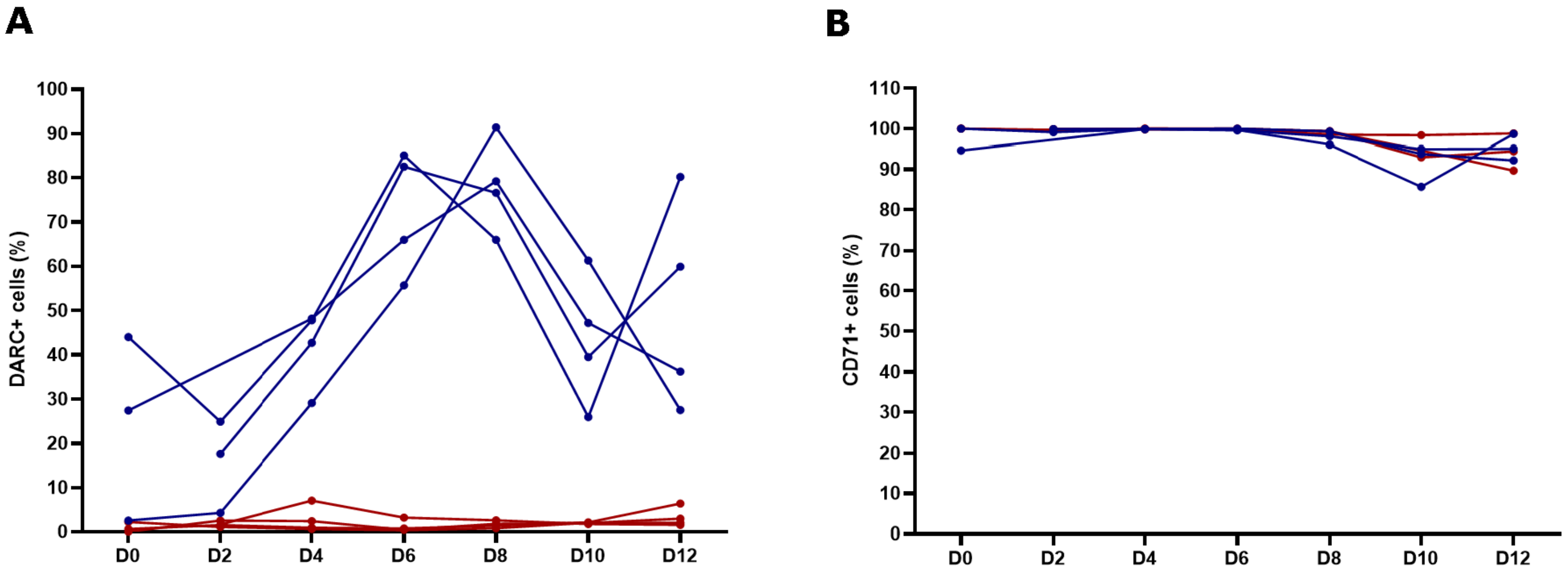

Figure S3 (Related to Figure 2): Expression of DARC and CD71 in Duffy-positive (DP) and Duffy-negative (DN) erythroblasts during terminal differentiation. A. The graphs represent the percentage of GPA+ DARC+ cells in four DP (*DP001, DP002, DP003, DP004*, blue lines) and four DN (*DN001, DN002, DN003, DN005*, red lines) donors at D0, D2, D4, D6, D8, D10 and D12. **B.** The graphs represent the percentage of GPA+ CD71+ cells in three DP (*DP002, DP003, DP004*, blue lines) and three DN (*DN002, DN003, DN005*, red lines) donors at D0, D2, D4, D6, D8, D10 and D12.

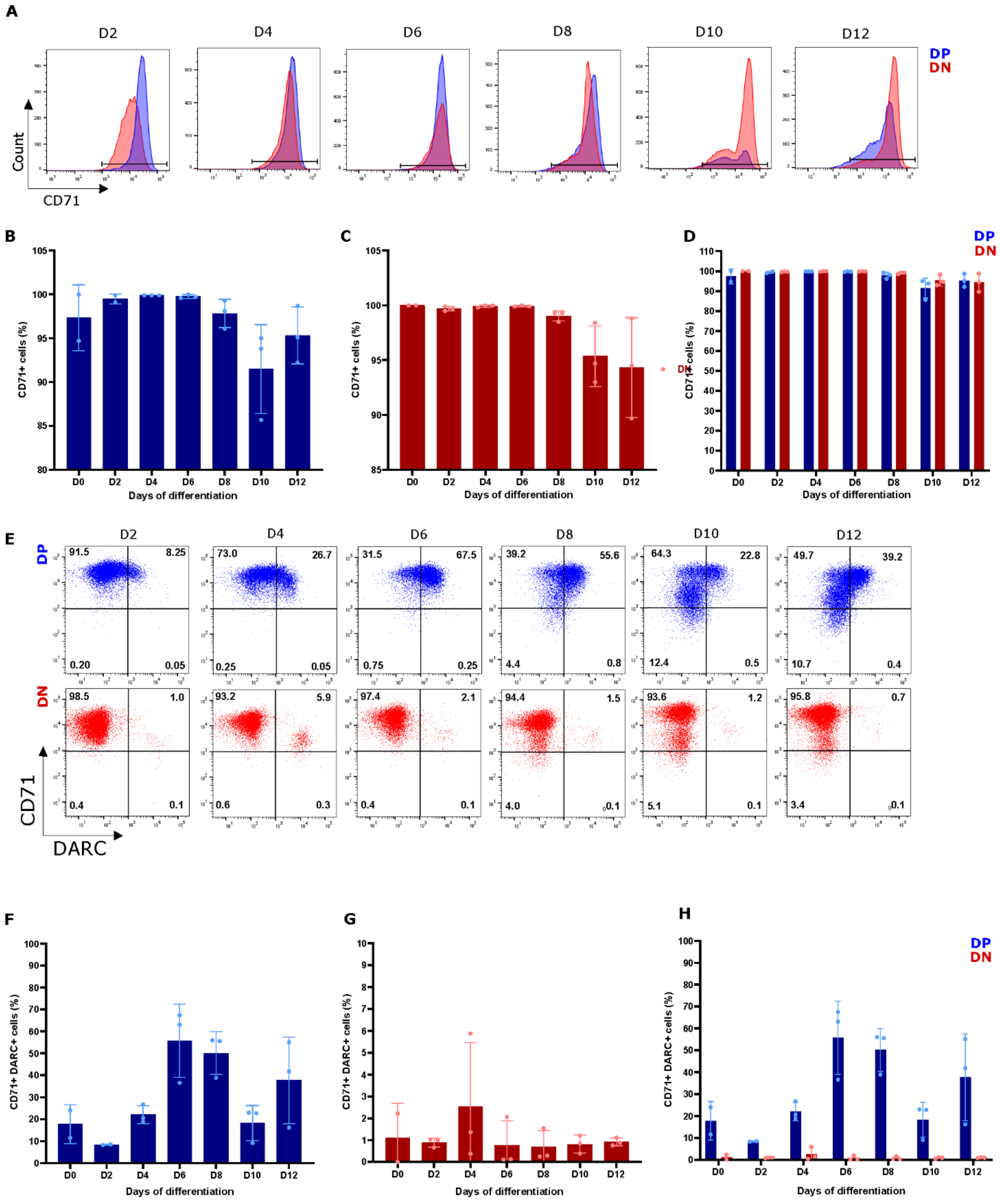

Figure S4 (Related to Figure 2 and Figure 3): Correlation between the expression of DARC and CD71 by Duffy-positive (DP) and Duffy-negative (DN) erythroblasts during terminal differentiation. A. The representative histograms show the percentage of GPA+ CD71+ cells in one DP (*DP004*) and one DN (*DN005*) donors at D0, D2, D4, D6, D8, D10 and D12. **B-D.** The bar charts represent the percentage of GPA+ CD71+ cells in three DP (*DP002, DP003, DP004*, blue bars)

and three DN (*DN002, DN003, DN005*, red bars) donors at D0, D2, D4, D6, D8, D10, and D12 (mean ± SD). **E.** A representative graph showing the distribution of GPA+ cells in one DP (*DP004*) and one DN (*DN005*) donors with respect to the expression of DARC (x-axis) and CD71 (y-axis) at D2, D4, D6, D8, D10 and D12**. F-H.** The bar charts represent the percentage of GPA+ CD71+ DARC+ cells in three DP (*DP002, DP003, DP004,* blue bars) and three DN (*DN002, DN003, DN005*, red bars) donors at D2, D4, D6, D8, D10, and D12 (mean ±SD).

A

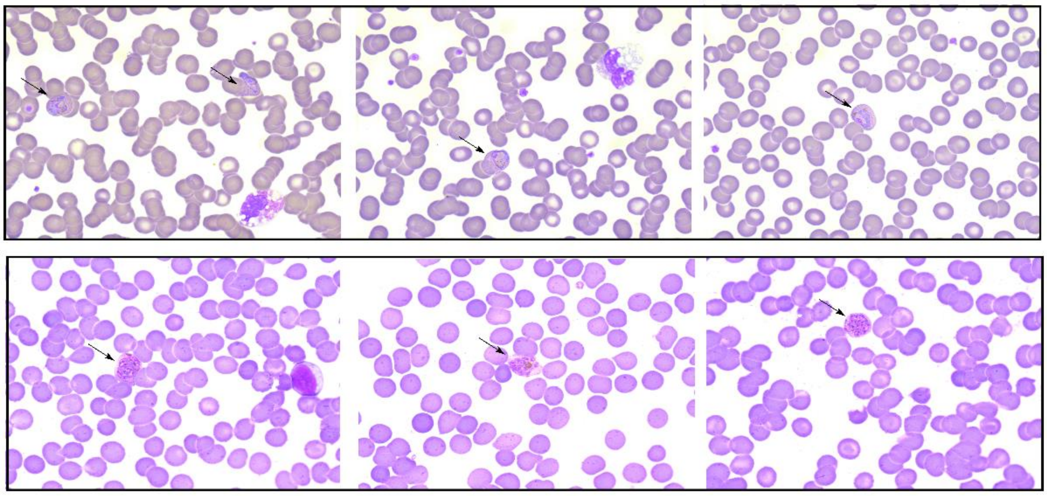

B

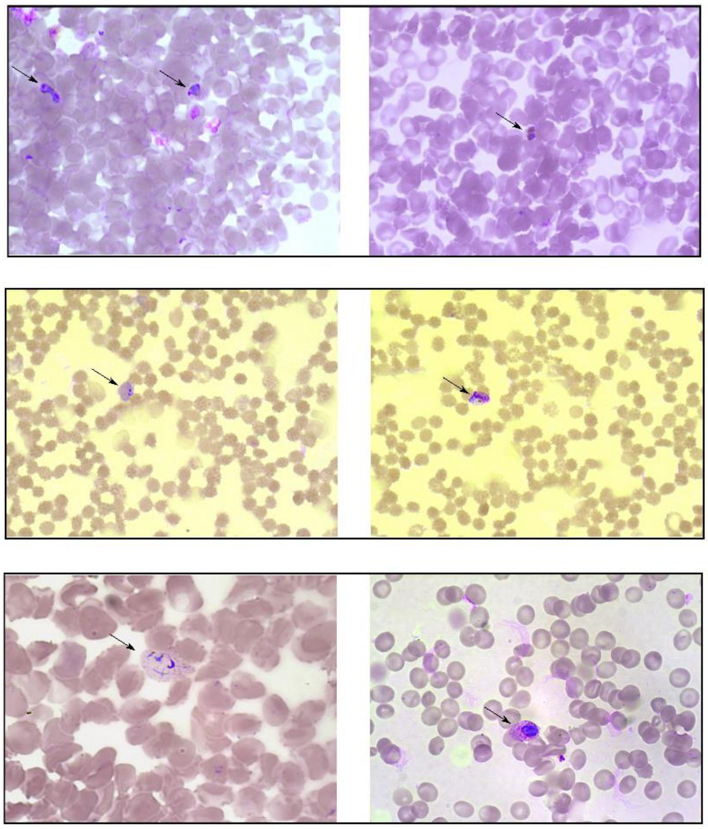

Figure S5 (Related to Figure 4): *P. vivax* parasites from Malagasy and Ethiopian cryoisolate blood samples after thawing and maturation (light microscopy). A. May-Grünwald Giemsa staining showing two Malagasy *P. vivax* isolates, respectively *MDZV02005* (first-line panel) and *MDZV02004* (second-line panel), after thawing. **B.** May-Grünwald Giemsa staining showing one Ethiopian *P. vivax* isolate (*MC4038*, first-line panel) and two Malagasy *P. vivax* isolates (*MDZV02004* and *MDZV02005*, second and third- line panel), after thawing and *in vitro* maturation for 24-30h.

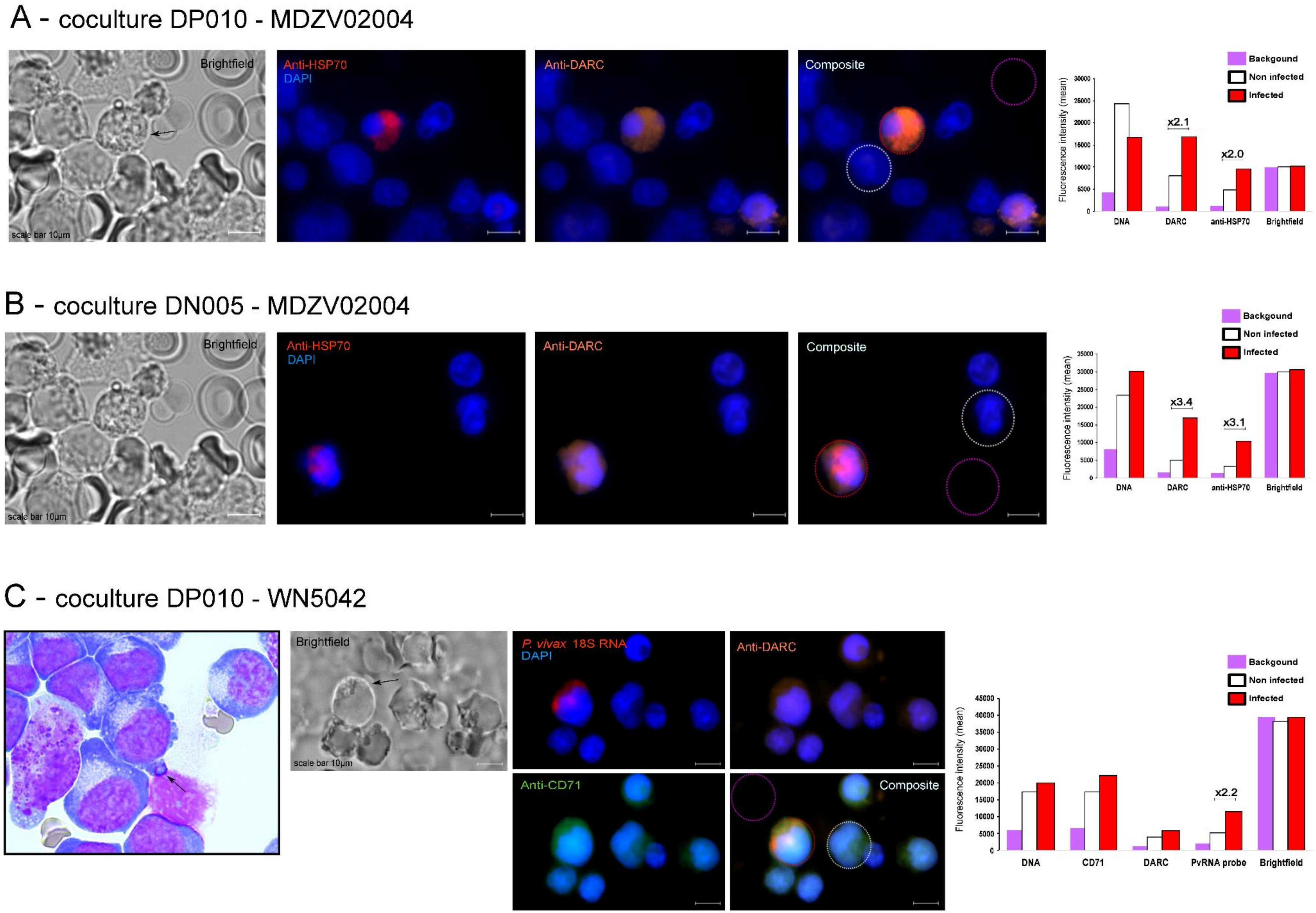
A - coculture DP010 - MDZV02004
Brightfield
scale bar 10µm
Anti-HSP70
DAPI
Anti-DARC
Composite
Backgound
Non infected
Infected
Fluorescence intensity (mean)
x2.1
x2.0
DNA
DARC
anti-HSP70
Brightfield
B - coculture DN005 - MDZV02004
Brightfield
scale bar 10µm
Anti-HSP70
DAPI
Anti-DARC
Composite
Backgound
Non infected
Infected
Fluorescence intensity (mean)
x3.4
x3.1
DNA
DARC
anti-HSP70
Brightfield
C - coculture DP010 - WN5042
Brightfield
scale bar 10µm
P. vivax 18S RNA
DAPI
Anti-DARC
Anti-CD71
Composite
Backgound
Non infected
Infected
Fluorescence intensity (mean)
x2.2
DNA
CD71
DARC
PvRNA probe
Brightfield

Figure S6 (Related to Figure 4): Additional *P. vivax* infections of Duffy-positive (DP) and Duffy-negative (DN) erythroblasts with Malagasy and Ethiopian *P. vivax* isolates (fluorescence microscopy). A to C. After 24h of co-culture, *P. vivax* parasites were observed on May Grunwald-Giemsa-stained slides by light microscopy and fluorescence microscopy [Brighfield + DAPI (blue)/RNA 18S FISH probe (red) + anti-DARC antibody (orange) + anti-CD71 antibody (green) or Brighfield + DAPI (blue)/anti-HSP70 antibody (red) + anti-DARC antibody (orange), objective 63X]. The bar graphs represent the mean fluorescence intensity (MFI) of the background (purple), non-infected cells (white), and P. vivax-infected cells (red) detected by fluorescence microscopy [DAPI (DNA, blue), RNA 18S FISH probe (red), anti-HSP70 antibody (red or green), anti-DARC antibody (orange), and anti-CD71 antibody (green)]. A scale bar is shown on each image. Additional details are provided in the Method details section and Table S1. **A.** *Co-culture DP010 - MDZV02004*. DP erythroblasts (at D9 of terminal differentiation) infected with a Malagasy *P. vivax* isolate. **B.** *Co-culture DN005 - MDZV02004*. DN erythroblasts (at D9 of terminal differentiation) infected with the same Malagasy *P. vivax* isolate. **C.** *Co-culture DP010 – WN5042*. DP erythroblasts (at D9 of terminal differentiation) were infected with Ethiopian *P. vivax* isolate and observed using light microscopy (mature ring stage) and fluorescence microscopy.

A - coculture DN005 - WN5042

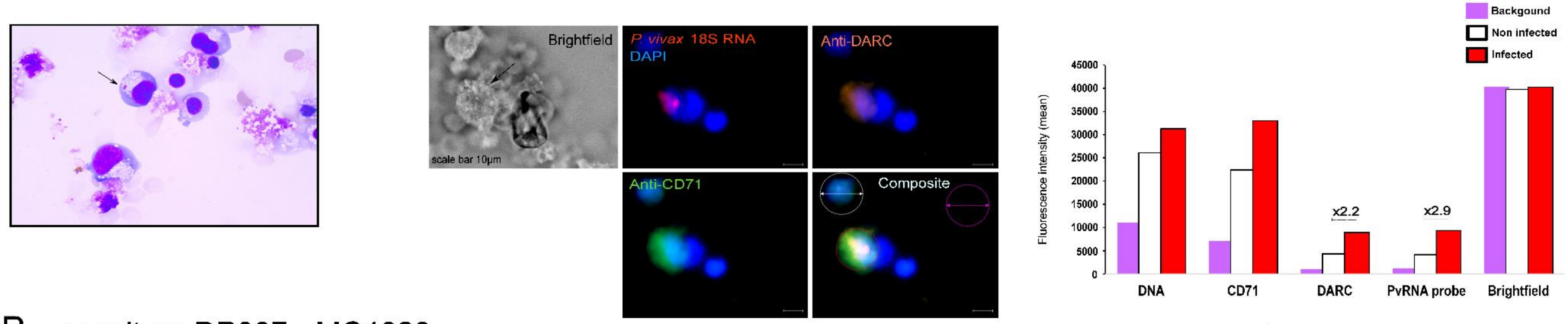

B - coculture DP007 - MC4023

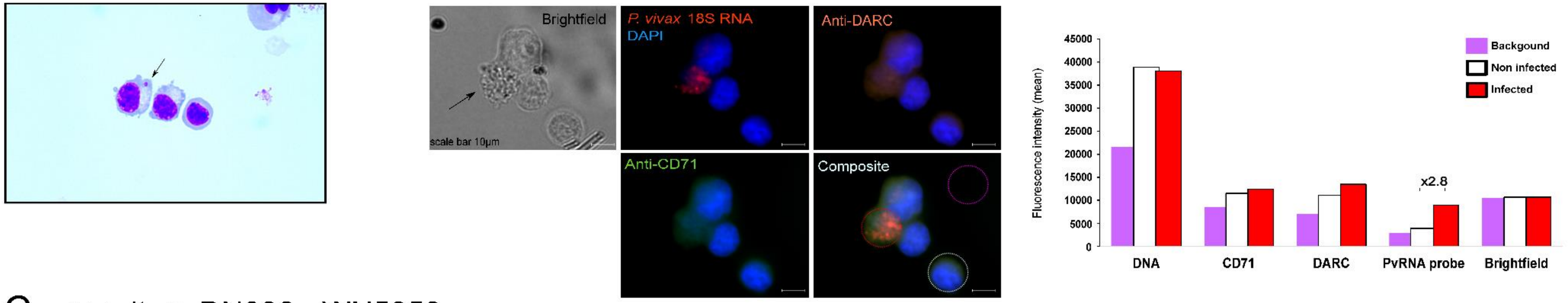

C - coculture DN006 - WN5050

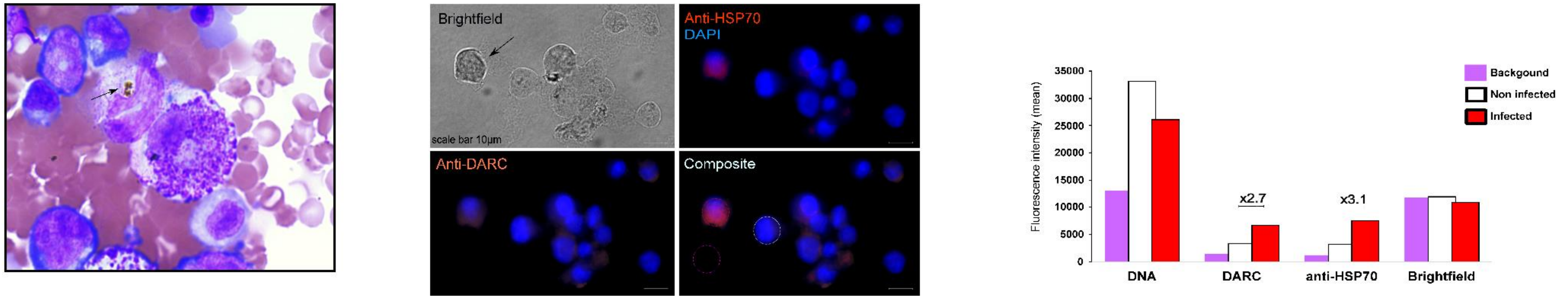

Figure S7 (Related to Figure 4): Additional *P. vivax* infections of DP and DN erythroblasts with the Ethiopian *P. vivax* isolates (light and fluorescence microscopy). A to C. After 48h of co-culture, *P. vivax* parasites were observed on May Grunwald-Giemsa-stained slides by light microscopy and fluorescence microscopy [Brightfield + DAPI (blue)/RNA 18S FISH probe (red) + anti-DARC antibody (orange) + anti-CD71 antibody (green) or Brightfield + DAPI (blue)/anti-HSP70 antibody (red) + anti-DARC antibody (orange), objective 63X]. The bar graphs represent the mean fluorescence intensity (MFI) of the background (purple), non-infected cells (white), and *P. vivax-i*nfected cells (red) detected by fluorescence microscopy [DAPI (DNA, blue), RNA 18S FISH probe (red), anti-HSP70 antibody (red or green), anti-DARC antibody (orange), and anti-CD71 antibody (green)]. A scale bar is shown on each image. Additional details are provided in the Method details section and Table S1. **A.** *Co-culture DN005 – WN5042*. DN erythroblasts (at D9 of terminal differentiation) were infected with Ethiopian *P. vivax* isolate by light microscopy (ring stage) and fluorescence microscopy. **B.** *Co-culture DP007 – MC4023.* DP erythroblasts (at D9 of terminal differentiation) were infected with Ethiopian *P. vivax* isolate and observed using light microscopy (mature stage with hemozoin) and fluorescence microscopy. **C.** *Co-culture DN006 – WN5050*. DN erythroblasts (at D9 of terminal differentiation) were infected with Ethiopian *P. vivax* isolate and observed by light microscopy (mature stage with hemozoin) and fluorescence microscopy.
