# Supplementary material for: Unveiling P. vivax invasion pathways in Duffy-negative individuals
Source: Cell Host Microbe. 2023 Dec 13;31(12):2080–2092.e5. doi: 10.1016/j.chom.2023.11.007 (PMC10727064; doi:10.1016/j.chom.2023.11.007)
Supplement: Document S2. Article plus supplemental information [file mmc3.pdf]

Clinical and Translational Report
Cell Host & Microbe

Unveiling *P. vivax* invasion pathways in Duffy-negative individuals

Graphical abstract

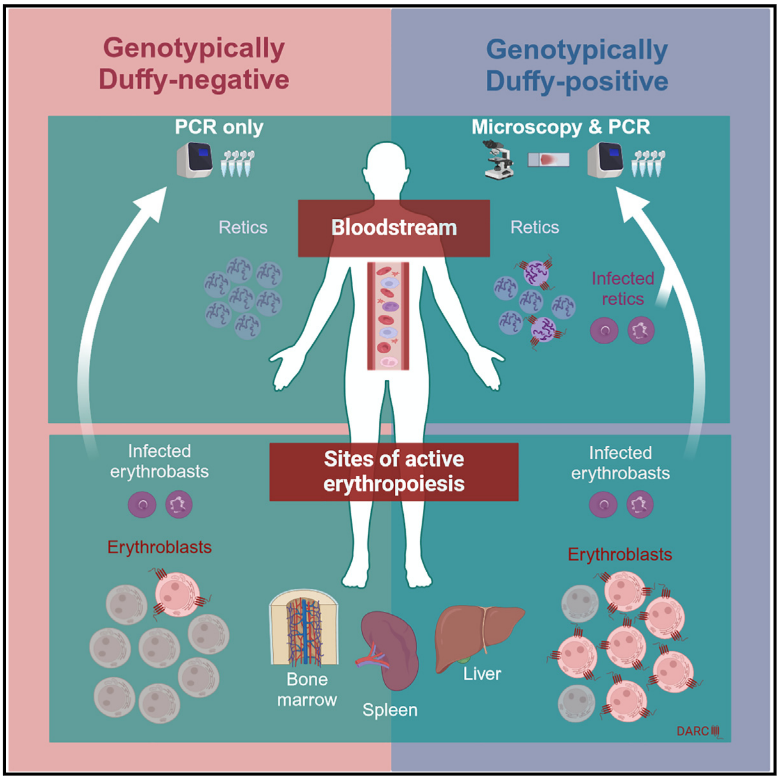

Authors

Isabelle Bouyssou, Sara El Hoss, Cécile Doderer-Lang, ..., Chetan E. Chitnis, John Strouboulis, Didier Ménard

Correspondence

sara.el_hoss@kcl.ac.uk (S.E.H.), john.strouboulis@kcl.ac.uk (J.S.), dmenard@pasteur.fr (D.M.)

In brief

P. vivax invades reticulocytes through Duffy-binding protein and DARC, a protein lacking in Duffy-negative Africans. We show that a subset of Duffy-negative erythroblasts transiently express DARC at erythropoiesis sites and can be infected by *P. vivax*, suggesting that deep *P. vivax* infection may be under-recognized as a health concern in Africa.

Highlights

- African Duffy-negative (DN) individuals are not fully resistant to vivax malaria
- A subset of DN erythroblasts express DARC during terminal erythroid differentiation
- *P. vivax* can invade DARC+ DN erythroblasts, regardless of their origin
- Deep *P. vivax* infection may be an unconsidered health problem in sub-Saharan Africa

Bouyssou et al., 2023, Cell Host & Microbe *31*, 2080–2092
December 13, 2023 © 2023 The Author(s). Published by Elsevier Inc.
https://doi.org/10.1016/j.chom.2023.11.007
CellPress

CellPress
OPEN ACCESS

Cell Host & Microbe

Clinical and Translational Report

Unveiling *P. vivax* invasion pathways in Duffy-negative individuals

Isabelle Bouyssou,[1,2,3,12] Sara El Hoss,[4,12,*] Cécile Doderer-Lang,[5] Matthieu Schoenhals,[6] Lova Tsikiniaina Rasoloharimanana,[6] Inès Vigan-Womas,[7] Arsène Ratsimbasoa,[8] Andargie Abate,[9] Lemu Golassa,[9] Solenne Mabilotte,[5] Pascal Kessler,[10] Micheline Guillotte-Blisnick,[3] Francisco J. Martinez,[3] Chetan E. Chitnis,[3] John Strouboulis,[4,*] and Didier Ménard[1,3,5,11,13,*]
[1]Malaria Genetics and Resistance Unit, INSERM U1201, Institut Pasteur, Université Paris Cité, 75015 Paris, France
[2]École Doctorale ED515 "Complexité du Vivant", Sorbonne Université, 75005 Paris, France
[3]Malaria Parasite Biology and Vaccines Unit, Institut Pasteur, Université Paris Cité, 75015 Paris, France
[4]Red Cell Haematology Laboratory, Comprehensive Cancer Centre, School of Cancer and Pharmaceutical Sciences, King's College London, London SE5 9NU, UK
[5]Institute of Parasitology and Tropical Diseases, UR7292 Dynamics of Host-Pathogen Interactions, Université de Strasbourg, 67000 Strasbourg, France
[6]Immunology of Infectious Diseases, Institut Pasteur of Madagascar, Antananarivo 101, Madagasca
[7]Institut Pasteur de Dakar, Dakar 220, Senegal
[8]Faculté de Médecine, Université de Fianarantsoa, Fianarantsoa 301, Madagascar
[9]Aklilu Lemma Institute of Pathobiology, Addis Ababa University, PO Box 1176, Addis Ababa, Ethiopia
[10]Centre de Recherche en Biomédecine de Strasbourg, Université de Strasbourg, 67000 Strasbourg, France
[11]Laboratory of Parasitology and Medical Mycology, CHU Strasbourg, 67000 Strasbourg, France
[12]These authors contributed equally
[13]Lead contact
*Correspondence: sara.el_hoss@kcl.ac.uk (S.E.H.), john.strouboulis@kcl.ac.uk (J.S.), dmenard@pasteur.fr (D.M.)
https://doi.org/10.1016/j.chom.2023.11.007

SUMMARY

Vivax malaria has long been thought to be absent from sub-Saharan Africa owing to the high proportion of individuals lacking the Duffy antigen receptor for chemokines (DARC) in their erythrocytes. The interaction between *P. vivax* Duffy-binding protein (*Pv*DBP) and DARC is assumed to be the main pathway used by merozoites to invade reticulocytes. However, the increasing number of reports of vivax malaria cases in genotypically Duffy-negative (DN) individuals has raised questions regarding the *P. vivax* invasion pathway(s).

Here, we show that a subset of DN erythroblasts transiently express DARC during terminal erythroid differentiation and that *P. vivax* merozoites, irrespective of their origin, can invade DARC+ DN erythroblasts. These findings reveal that a large number of DN individuals may represent a silent reservoir of deep *P. vivax* infections at the sites of active erythropoiesis with low or no parasitemia, and it may represent an underestimated biological problem with potential clinical consequences in sub-Saharan Africa.

INTRODUCTION

More than one-third of the world's population is affected by vivax malaria, an acute debilitating disease caused by *Plasmodium vivax* and transmitted by female *Anopheles* mosquitoes. In 2021, there were an estimated five million vivax malaria cases worldwide, mainly in Asia, Southeast Asia, South America, the Western Pacific, Eastern Mediterranean regions, Eastern Africa, and Southern Africa.[1]

For a long time, vivax malaria was thought to be absent in sub-Saharan Africa. This was based on the clinical and field studies conducted in the 1940s, which showed natural resistance to vivax malaria in Africans and Afro-Americans.[2–5] Later, Duffy blood groups were identified,[6,7] and it was demonstrated that the absence of the Duffy antigen receptor for chemokines (DARC) on the surface of human erythrocytes, caused by a single-point mutation in the GATA-1 promoter sequence of *ackr1*−, was associated with natural resistance to vivax malaria.[8] Pioneering studies in the 1980s and the 1990s identified the *P. vivax* Duffy-binding protein (*Pv*DBP) as a specific ligand for the N-terminal extracellular domain (ECD1) of the host DARC and confirmed that the interaction between *Pv*DBP and DARC is critical for reticulocyte invasion.[9,10] More recently, additional studies have revealed CD71 (transferrin receptor [TfR1]) and CD98 as the respective cogent receptors for *P. vivax* reticulocyte-binding protein 2b (*Pv*RBP2b) and *P. vivax* reticulocyte-binding protein 2a (*Pv*RBP2a).[11–13] Together, these findings led to a scientific paradigm whereby *P. vivax* recognizes

 © 2023 The Author(s). Published by Elsevier Inc.
This is an open access article under the CC BY license (http://creativecommons.org/licenses/by/4.0/).

Table 1. Duffy genotyping of healthy blood donors

Blood donor	Mutation GATA-1	Mutation Fy a/b	Mutation Fy Codon 49	Codon 89	Codon 100	Phenotype	Use in this study
DP001	TCT	GAT (b)	GCC	CGC	ACA	Fy (a−b+)	characterization
DP002	TCT	GAT (b)	GCC	CGC	GCA/ACA	Fy (a−b+)	characterization
DP003	TCT	GAT (b)/GGT (a)	GCC	CGC	GCA	Fy (a+b+)	characterization
DP004	TCT	GAT (b)/GGT (a)	GCC	CGC	GCA/ACA	Fy (a+b+)	characterization
DP007	TCT	GAT (b)/GGT (a)	GCC	CGC	GCA	Fy (a+b+)	characterization and invasion
DP010	TCT	GAT (b)/GGT (a)	GCC	CGC	GCA/ACA	Fy (a+b+)	invasion
DN001	CCT	GAT (b)	GCC	CGC	GCA	Fy (a−b−)	characterization
DN002	CCT	GAT (b)	GCC	CGC	GCA	Fy (a−b−)	characterization
DN003	CCT	GAT (b)	GCC	CGC	GCA	Fy (a−b−)	characterization
DN005	CCT	GAT (b)	GCC	CGC	GCA	Fy (a−b−)	characterization and invasion
DN006	CCT	GAT (b)	GCC	CGC	GCA	Fy (a−b−)	characterization and invasion

reticulocytes through interactions with *Pv*RBP2b-CD71 and *Pv*RBP2a-CD98 and invades genotypically Duffy-positive (DP) DARC+ reticulocytes through interactions between *Pv*DBP and DARC.[14]

However, this paradigm has been called into question. With the advent of nucleic acid amplification tests (NAATs), an increasing number of *P. vivax* infections have been reported in genotypically Duffy-negative (DN) sub-Saharan African populations. In most cases, these infections are characterized by submicroscopic or very low levels of parasitemia.[15–18] In parallel, *P. vivax* has been shown to preferentially invade CD71$^+$ immature red blood cells (i.e., reticulocytes) and erythroid precursors, found primarily in extravascular erythropoietic tissues, such as the bone marrow,[19–27] spleen,[28–30] and tissues where extramedullary erythropoiesis can occur.[31] These observations demonstrate that anatomic sites of active erythropoiesis provide niches where pathogenic *P. vivax* biomass increases may occur, implying that vivax infections are more common than suggested by the presence of vivax parasites in peripheral blood.[15]

Here, we investigated the infection of genotypically DP and DN erythroblasts with *P. vivax* merozoites *in vitro* to explore and identify host receptors that *P. vivax* uses to invade erythroblasts. We performed *in vitro* erythropoiesis assays of genotypically DN and DP erythroid progenitors to critically evaluate terminal erythroid differentiation and CD71 and DARC expression profiles over time. We then conducted *in vitro* invasion assays to further investigate whether DARC expressed on genotypically DN erythroblasts is functional for *P. vivax* invasion. Our key findings were that a subset of genotypically DN erythroblasts transiently express DARC during terminal erythroid differentiation and that this subpopulation is susceptible to *P. vivax* merozoite invasion, providing biological evidence that *P. vivax* can invade a subset of DN erythroblasts that transiently express DARC as a functional receptor for invasion.

RESULTS

Genotypically DP and DN erythroid progenitors show similar kinetics of differentiation

To determine whether genotypically DN erythroid progenitors have similar erythroid differentiation kinetics to genotypically DP erythroid progenitors, we isolated CD34+ hematopoietic stem and progenitor cells (HSPCs) from the peripheral blood of four DP (DP001, DP002, DP003, and DP004) and four DN (DN001, DN002, DN003, and DN005) healthy donors (Table 1). CD34+ cells were expanded and differentiated *in vitro* (Figure 1A). The kinetics of terminal erythroid differentiation, apoptosis rates, and enucleation yields were monitored every other day for 12 days using optical microscopy and flow cytometry. Both DP and DN erythroid progenitors presented the expected pattern, with proerythroblasts maturing into early basophilic erythroblasts, late basophilic erythroblasts, polychromatic erythroblasts, and orthochromatic erythroblasts.[32]

The mean percentages of DP and DN erythroblasts at the orthochromatic stage were 0.08% (n = 3, confidence interval 95%: 0%–0.44%) and 0% (n = 3) on day 0, 19.8% (n = 4, confidence interval 95%: 0%–45.2%) and 6.7% (n = 3, confidence interval 95%: 0%–18.9%) on day 6, and 85% (n = 4, confidence interval 95%: 66.5%–100%) and 62.1% (n = 4, confidence interval 95%: 28.5%–95.7%) on day 12, respectively (Figures 1B and 1C). No significant difference in the kinetics of terminal erythroid differentiation of DP and DN erythroblasts was observed over time (analysis of covariance [ANCOVA], p = 0.4; proerythroblasts, p = 0.2; early basophilic erythroblasts, p = 0.6; late basophilic erythroblasts, p = 0.9; polychromatic erythroblasts, p = 0.08 for orthochromatic erythroblasts) (Figures 1C and 1D). However, we found considerable inter-individual variation in DP and DN donors. The relative standard deviation (RSD) of the proportions of erythroblast stages varied from 6% to 180% for DP erythroblasts, and from 9% to 180% for DN erythroblasts (Figures S1A and S1B). No significant difference in variability was detected between DP and DN erythroblasts (p = 0.15, Mann-Whitney test).

The enucleation rate was also monitored on day 8 (n = 3, mean: 4.8%, confidence interval 95%: 0%–18.4% for DP and n = 3, mean: 1.2%, confidence interval 95%: 0%–4.3% for DN) and on day 10 (n = 4, mean: 28.3%, confidence interval 95%: 0%–58.4% for DP and n = 4, mean: 11.9%, confidence interval 95%: 0%–25.0% for DN). Although we found no significant difference in the proportion of enucleated cells between DN and DP erythroblasts (ANCOVA, p = 0.14), we observed a slight delay in the enucleation of DN erythroblasts on day 10 (Figures S2A and S2B). Finally, low and similar proportions of Glycophorin

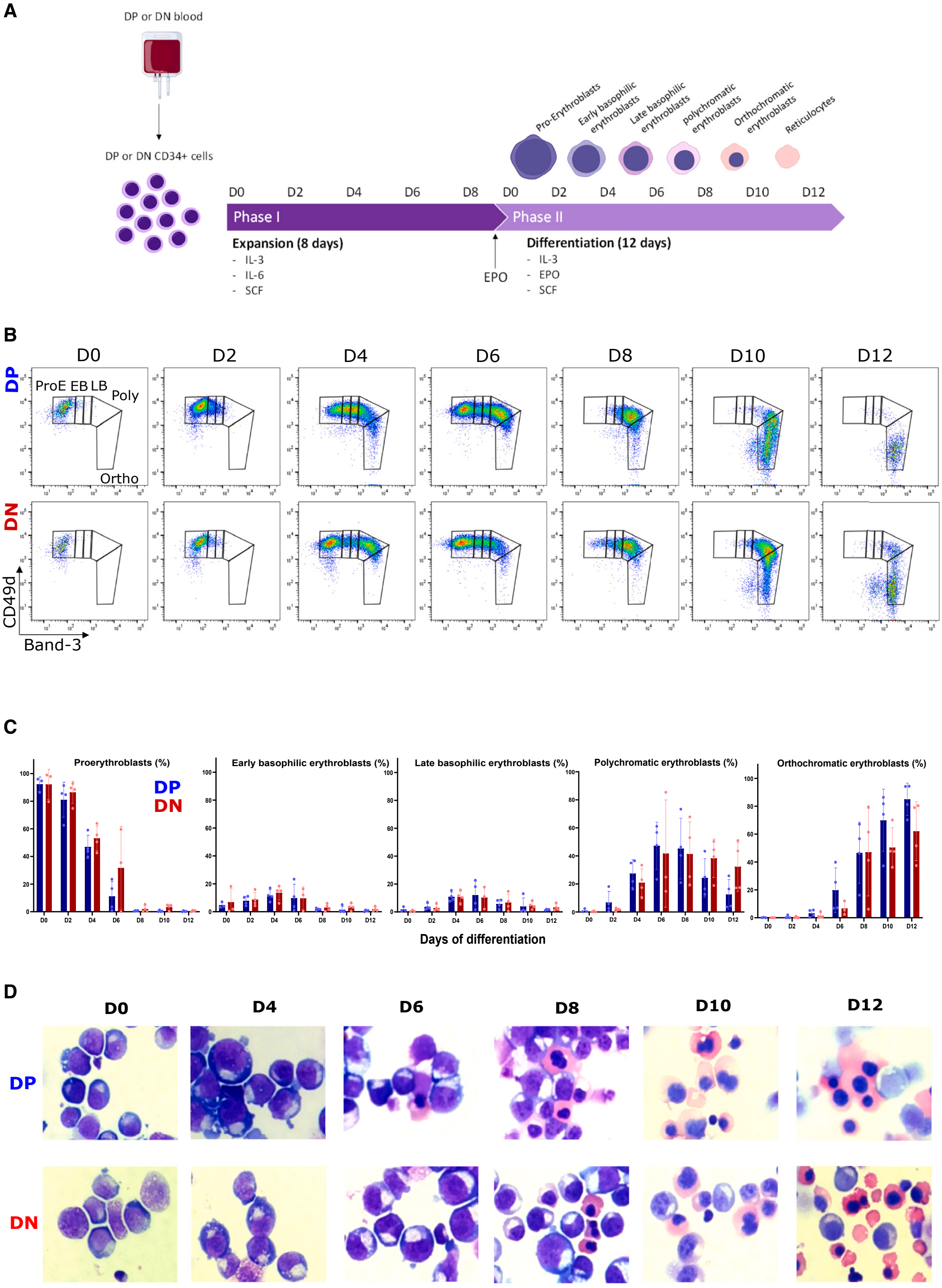

Figure 1. Genotypically Duffy-positive (DP) and Duffy-negative (DN) erythroid progenitors show similar kinetics of differentiation

Figure360▷ For a Figure360 author presentation of this figure, see https://doi.org/10.1016/j.chom.2023.11.007.

(legend continued on next page)

A-positive (GPA+) Annexin V+ cells (reflecting apoptosis) were noticed in both DP and DN erythroblasts, indicating that they survived at similar rates during *in vitro* differentiation (Figures S2C and S2D).

We conclude that both genotypically DP and DN erythroblasts have similar terminal erythroid differentiation kinetics.

A subset of genotypically DN erythroblasts transiently express DARC during terminal erythroid differentiation

To determine the proportions of genotypically DP and DN erythroblasts expressing DARC and CD71 during terminal erythroid differentiation, we quantified the DARC/CD71 expression profiles every other day for 12 days by flow cytometry. We observed that the mean percentage of DP erythroblasts expressing DARC among the four donors (DP001, DP002, DP003, and DP004) varied from 24.6% (n = 3, confidence interval 95%: 0%–76.4%) on day 0 (mostly proerythroblasts) to 78.3% (n = 4, confidence interval 95%: 61.7%–94.9%) on day 8 (mostly polychromatic and orthochromatic erythroblasts) and 50.9% (n = 4, confidence interval 95%: 13.0%–88.9%) on day 12 (mostly orthochromatic erythroblasts) (Figures 2A–2C, 2E, and S3A). The proportion of DARC+ DP erythroblasts did not differ significantly over time ($p = 0.07$, one-way analysis of variance) but was highly variable among the four donors. The RSD ranged from 13% (day 8) to 85% (day 0) (Figure S1C). More importantly, we observed that a subset of DN erythroblasts transiently expresses DARC. The mean percentage of DN erythroblasts expressing DARC (DN001, DN002, DN003, and DN005) was lower than that of DARC+ DP erythroblasts, varying from 1.0% (n = 3, confidence interval 95%: 0%–3.8%) on day 0 (mostly proerythroblasts) to 1.6% (n = 4, confidence interval 95%: 0.5%–2.8%) on day 8 (mostly polychromatic and orthochromatic erythroblasts) and 3.2% (n = 4, confidence interval 95%: 0%–6.7%) on day 12 (mostly orthochromatic erythroblasts) (Figures 2A–2E, and S3A). Interestingly, we noted that the subset of DN erythroblasts expressing DARC showed a higher MFI than that of DARC+ DP erythroblasts (Figures 2A and 2B). As observed with DARC+ DP erythroblasts, we found that the proportion of DARC+ DN erythroblasts did not differ significantly over time ($p = 0.7$, one-way analysis of variance) but was highly variable among the four donors, with RSD ranging from 7% (day 10) to 120% (day 0) (Figure S1C).

We further confirmed the expression of DARC in DP and DN erythroblasts and donor erythrocytes by western blot analysis of cytoplasmic protein extracts. We detected differential expression of DARC in both DP and DN erythroblasts during the early stages of differentiation (Figures 3A and 3B). In DN cells, DARC expression was highly variable among the donors (DN001, DN002, DN003, and DN005), whereas DARC expression remained less variable among DP donor cells (DP001, DP003, DP004, and DP007) throughout differentiation. The relative amount of DARC protein was significantly lower in DN erythroblasts at days 10 and 12 of terminal differentiation compared with DP erythroblasts ($p = 0.03$, Mann-Whitney test) (Figures 3A and 3B). The DARC expression pattern in DN cells during *in vitro* erythroid differentiation was consistent with the observation that DN erythrocytes from the peripheral blood of the donor were not DARC+ (Figure 3A; red blood cells from DN005 donor).[33] This finding was supported by fluorescence microscopy, which showed that only a few DN erythroblasts were DARC+ at day 9 terminal differentiation (Figure 3C).

Hence, we conclude from these experiments that DN erythroblasts clearly express DARC during erythroid differentiation, albeit at lower levels compared with DP erythroblasts.

We observed similar CD71 expression profiles in both DP and DN erythroblasts (Figures S3B and S4A–S4D). The mean percentage of DP erythroblasts expressing CD71 was 97.3% (n = 3, confidence interval 95%: 63.7%–100%) on day 0 and 95.3% (n = 3, confidence interval 95%: 87.2%–100%) on day 12, similar to the mean percentage of DN erythroblasts expressing CD71 (n = 3, mean: 100%, confidence interval 95%: 100% on day 0 and n = 3, mean: 94.3%, confidence interval 95%: 83.0%–100% on day 12). There was a low level of variability within DP and DN donors (RSD < 5%) (Figure S1D). As a result, the mean proportion of DP erythroblasts expressing both DARC and CD71, the two major surface receptors required for *P. vivax* invasion, was highly variable, ranging from 8.4% (n = 3, confidence interval 95%: 6.6%–10.1%) on day 2 to 55.7% (n = 3, confidence interval 95%: 14.2%–97.3%) on day 6. This proportion remained higher than that of a subset of DARC+/CD71+ DN erythroblast (ranging from 0.7%, n = 3, confidence interval 95%: 0%–2.5% at day 8 to 2.5%, n = 3, confidence interval 95%: 0%–9.8% at day 4) (Figures S4E–S4H).

P. vivax merozoites can invade DN erythroblasts during terminal erythroid differentiation

To determine whether the subset of genotypically DN erythroblasts expresses functional DARC and CD71, the receptors for *Pv*DBP and *Pv*RBP2b that may allow *P. vivax* merozoite invasion, we isolated CD34+ HSPCs from the peripheral blood of two additional DP (DP007 and DP010) and two DN (DN005 and DN006) healthy donors (Table 1). CD34+ cells underwent expansion and differentiation *in vitro* (Figure 4A). Both DP and DN erythroid progenitors differentiated and reached the terminal differentiation stage, as described above. In both DP and DN erythroblasts, the expression profiles of DARC and CD71 were consistent with our previous findings. At day 7 of terminal differentiation, we thawed and cultured parasite isolates obtained from two Malagasy patients and seven

(A) The diagram illustrates the two-phase *in vitro* culture system for the isolation of CD34+ cells followed by the expansion and differentiation of erythroid progenitors.

(B) Representative flow cytometry contour plot of GPA+ cells from one DP (*DP001*) and one DN (*DN001*) donor, showing the distribution of cell populations with respect to expression of Band-3 (x axis) and CD49d (y axis) at days 0, 2, 4, 6, 8, 10, and 12. ProE, proerythroblasts; EB, early basophilic erythroblasts; LB, late basophilic erythroblasts; Poly, polychromatic erythroblasts; Ortho, orthochromatic erythroblasts.

(C) Bar graphs representing the percentage of proerythroblasts, early basophilic, late basophilic, polychromatic, and orthochromatic erythroblasts within the GPA+ population in four DP (*DP001*, *DP002*, *DP003*, and *DP004*, blue bars) and four DN (*DN001*, *DN002*, *DN003*, and *DN005*, red bars) donors on days 0, 2, 4, 6, 8, 10, and 12 (mean ± standard deviation).

(D) May-Grunwald Giemsa staining showing erythroid cells from one DP (*DP001*) and one DN (*DN001*) donors at day 0 (mainly proerythroblasts), day 4 (basophilic erythroblasts), day 6 (basophilic erythroblasts), day 8 (polychromatic erythroblasts), day 10, and day 12 (orthochromatic erythroblasts).

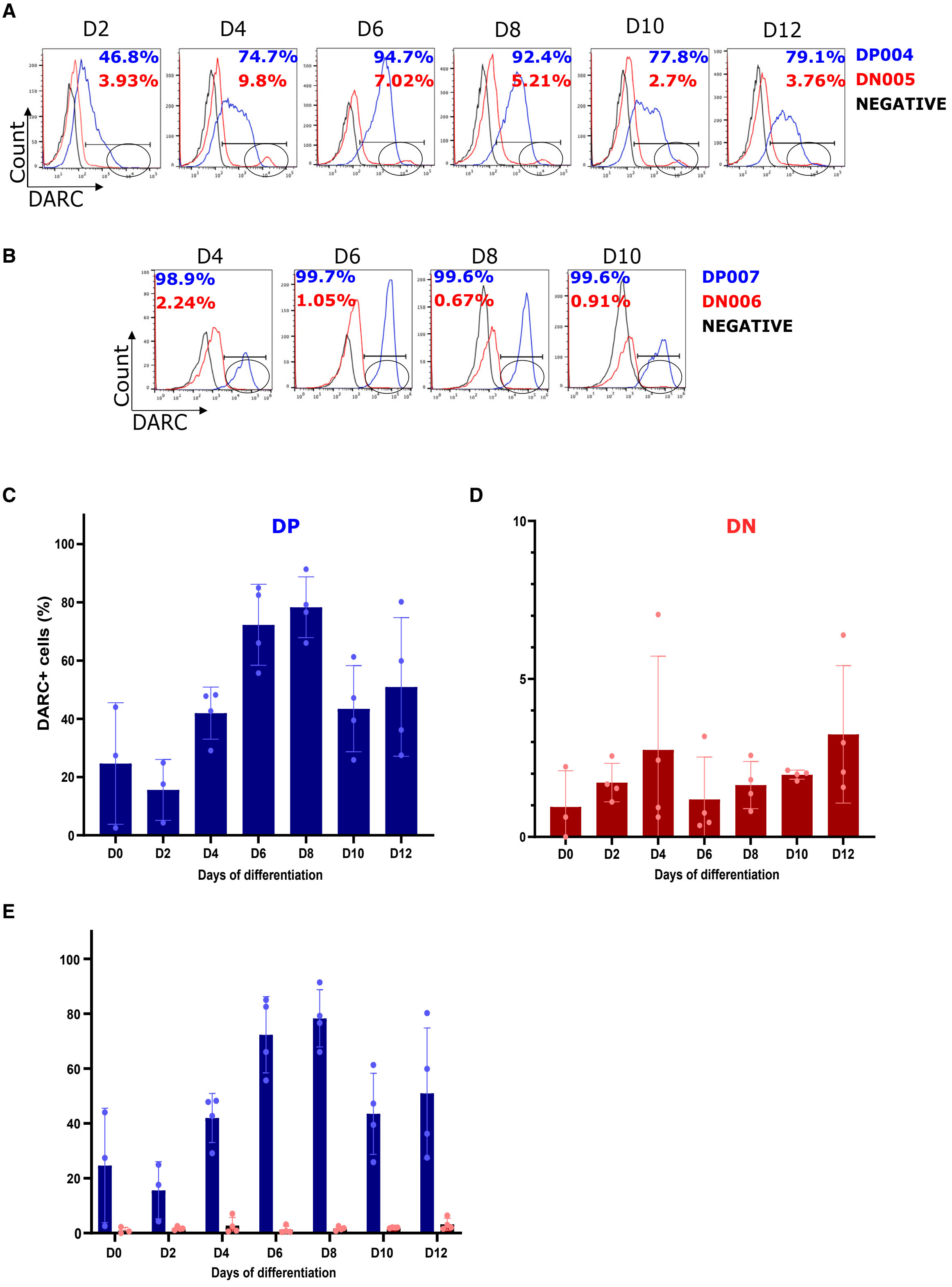

Figure 2. A subset of genotypically Duffy-negative (DN) erythroblasts express DARC during terminal erythroid differentiation (flow cytometry data)

Figure360▷ For a Figure360 author presentation of this figure, see https://doi.org/10.1016/j.chom.2023.11.007.

(A) A representative histogram showing the percentage of GPA+ DARC+ cells from one DP (*DP004*) and one DN (*DN005*) donor at days 2, 4, 6, 8, 10, and 12.

(legend continued on next page)

Ethiopian patients infected with *P. vivax* (Table S1; Figure 4A). After approximately 24–30 h of *in vitro* maturation (which corresponds to day 8 of terminal differentiation for DP and DN erythroblasts) (Figures S5A and S5B), each *P. vivax* parasite culture was co-cultured with either DP or DN erythroblasts for 24–48 h. We then assessed the infection of DP and DN erythroblasts by *P. vivax* using light microscopy. Approximately 50,000 cells were cytospun onto glass microscope slides and stained with May-Grunwald-Giemsa stain. Of the 22 co-cultures, we detected *P. vivax* infection in both DP and DN co-cultures (Table S1). We observed very few parasites, either at ring stages or at more mature stages, with large chromatin dots and hemozoin (Figures 4B–4G, S6C, and S7). No Schuffner's dots were observed in the infected erythroblasts.

We confirmed the infection of DP and DN erythroblasts with *P. vivax* parasites using fluorescence microscopy. About 3 × 10^6 erythroblasts per condition were stained with *P. vivax* 18S rRNA fluorescence in situ hybridization (FISH) probe, anti-*Plasmodium* HSP70, anti-DARC, and anti-CD71 antibodies. Of the 22 co-cultures (11 with DP and 11 with DN erythroblasts), *P. vivax* infections were detected in both DP and DN co-cultures (Figures 4B–4G, S6, and S7). Dual staining with the 18S rRNA FISH probe and anti-*Plasmodium* HSP70 antibody confirmed the presence of *P. vivax* parasites in DP and DN co-cultures (Figures 4B and 4C). Almost half of the *P. vivax* isolates invaded both DP and DN erythroblasts (5/11), 2/11 invaded neither, 3/11 invaded only DP erythroblasts, and 1/11 invaded only DN erythroblasts, suggesting that the ability to invade is critically dependent on the viability of the cryopreserved *P. vivax* parasites (Table S1). However, as only one to three infected erythroblasts were detected per invasion assay, we were unable to compare the number of *P. vivax*-infected cells in the invasion assays between DP and DN erythroblasts. As observed by light microscopy, both *P. vivax* parasites in DP and DN erythroblasts had a similar profile, in that we could observe the parasite nuclei close to the erythroblast nuclei, sometimes surrounded by signals corresponding to *the P. vivax* 18S rRNA probe and/or HSP70 monoclonal antibody (mAb), together with the expression of CD71 and DARC on the surface of the erythroblasts (Figures 4B–4G, S6, and S7). We found that all DP and DN erythroblasts infected with *P. vivax* were CD71+ and DARC+.

These results provide biological evidence that *P. vivax* can invade DN erythroblasts and that transiently expressed DARC is a potentially functional receptor for invasion.

DISCUSSION

Here, we provide key insights into *P. vivax* invasion pathways in genotypically DN patients by investigating the terminal erythroid differentiation of DN erythroid progenitors and their potential to express DARC and CD71 and present strong biological evidence that *P. vivax* merozoites, regardless of their origin, can invade DN erythroblasts.

The expression profile of DARC in DP erythroblasts was consistent with previous observations that approximately half of the erythroblasts expressed DARC on day 12.[34] Interestingly, we found that a small population of DN erythroblasts (<5% erythroblasts on day 12) also express DARC. This suggests that although a single-point mutation in the GATA-1 site of the *ackr1* gene promoter significantly reduces the number of DARC+ cells,[33] transient expression of DARC may occur in a small subpopulation of DN erythroid cells (~16-fold lower than the number of genotypically DP cells) and supports *P. vivax* infection. This observation was confirmed by the detection of DARC by western blotting in cytoplasmic protein extracts from DN erythroblasts but not in extracts from DN peripheral blood erythrocytes. Taken together, these data suggest that the GATA-1 binding motif of the *ackr1* promoter may be required for the sustained expression of DARC during erythroid differentiation in DP cells, whereas a single-point mutation in the GATA-1 motif in DN cells results in a decrease in the number of cells expressing DARC at terminal differentiation.

In vivo, the maturation of orthochromatic erythroblasts into reticulocytes involves various changes, including enucleation, hemoglobinization, remodeling of the membrane, and expression or depletion of certain proteins.[35] Here, we clearly show that DN erythroblasts are deficient in maintaining their ability to express DARC at the end of terminal erythroid differentiation when orthochromatic erythroblasts enucleate to produce reticulocytes or when reticulocytes migrate to peripheral blood and mature into erythrocytes. However, in this study, we were unable to provide conclusive evidence that the subset of DN erythroblasts expressing DARC gives rise to DN reticulocytes expressing DARC. Indeed, we cannot exclude the possibility that the threshold detection of the DARC protein by western immunoblotting, which is based on the measurement of total cytoplasmic proteins, may have failed to detect a small subset of DARC+ cells among the majority of DARC reticulocytes or erythrocytes due to the presence of large amounts of other proteins, such as hemoglobin.

Overall, we observed that the number of DP and DN erythroblasts expressing DARC during terminal erythroid differentiation varied widely between donors, probably due to the involvement of multiple regulatory elements controlling mRNA expression of the *ackr1* locus, among others, that may influence gene regulation. DARC expression profiles contrasted with CD71 expression profiles in both DP and DN erythroblasts, consistent with previous observations.[36] CD71 was highly expressed in erythroid precursors at all maturation stages and decreased as reticulocytes matured.[37–39] Given the specific tropism of *P. vivax* merozoites for CD71+ immature reticulocytes[13] and the importance of the *Pv*RBP2b-CD71 interaction in the recognition of DP reticulocytes prior to invasion,[11] our data confirmed that *P. vivax*

(B) A representative histogram showing the percentage of GPA+ DARC+ cells in erythroid cells from one DP (*DP007*) and one DN (*DN006*) donor at days 4, 6, 8, and 10. The oval shapes in (A) and (B) show DARC+ DN erythroid cells.

(C) Bar graph representing the percentage of GPA+ DARC+ cells in a total of four DP (*DP001*, *DP002*, *DP003*, and *DP004*) donors on days 0, 2, 4, 6, 8, 10, and 12 (mean ± standard deviation).

(D) Bar graph representing the percentages of GPA+ DARC+ cells in a total of four DN (*DN001*, *DN002*, *DN003*, and *DN005*) donors on days 0, 2, 4, 6, 8, 10, and 12 (mean ± standard deviation).

(E) A bar graph representing the percentage of GPA+ DARC+ cells in erythroid cells from four DP (*DP001*, *DP002*, *DP003*, and *DP004*) donors and four DN (*DN001*, *DN002*, *DN003*, and *DN005*) donors on days 0, 2, 4, 6, 8, 10, and 12 (mean ± standard deviation).

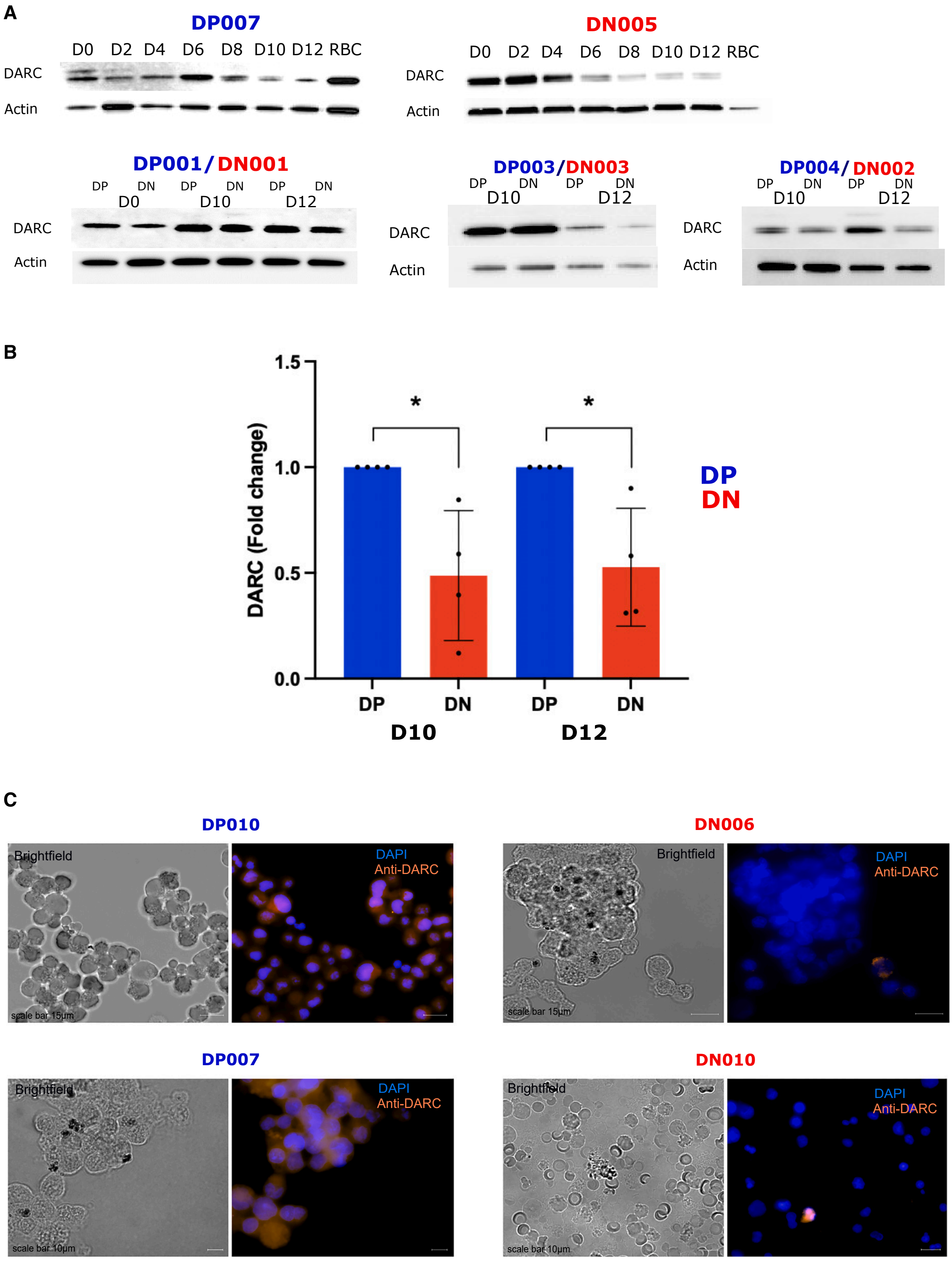

(legend on next page)

CellPress
OPEN ACCESS

infections are restricted to CD71+ erythroid progenitors, erythroblasts, and/or immature reticulocytes.

Since the development of functional invasion assays requires large numbers of erythroblasts, we decided to establish co-cultures on day 8 for terminal differentiation (Figure 4A). DP and DN erythroblasts were co-cultured with either Malagasy or Ethiopian merozoites of *P. vivax*. When DN erythroblasts were cocultured with *P. vivax* parasites, only DARC+/CD71+ DN erythroblasts were invaded by the parasites. These observations suggest that DN erythroblasts express functional DARC and that *P. vivax* merozoites can use the *Pv*DBP-DARC invasion pathway, irrespective of the origin and genetic background of the *P. vivax* strains. This was confirmed as there was no evidence of *P. vivax* invasion of erythroid cells that were not positive for DARC. Therefore, this finding suggests that *P. vivax* has not evolved an alternative invasion pathway to overcome Duffy-negativity.[40,41] However, definitive evidence through the use of an anti-DARC mAb to prevent erythroblast invasion by *P. vivax* is not provided in our study. These additional experiments were not performed due to the low number of infected cells per assay (approximately 1–3 infected cells) and the inability to accurately quantify the difference in the number of infected cells between the two culture conditions (with and without mAb anti-DARC).

Overall, the expression of DARC assessed in erythroid progenitors from DN donors may confirm the accumulating evidence of *P. vivax* endemicity across the DN population and provide evidence that *P. vivax* may be a prominent biological problem in sub-Saharan Africa.[15,42,43] The results obtained here suggest that the biomass of *P. vivax* shifts away from the peripheral blood into the extravascular spaces of the bone marrow and other tissues, where immature reticulocytes can form or accumulate. This echoes old and recent reports of *P. vivax* presence in the bone marrow and the spleen, which have been observed by autopsy, aspiration, biopsy, or acute infection following bone marrow transplantation.[15,30] Therefore, anatomic sites of active erythropoiesis, including bone marrow, spleen, liver, and other tissues in which extramedullary hematopoiesis can occur (as seen in many pathologies such as chronic and deep anemia), may represent a hidden reservoir of *P. vivax* parasites,[19–31] suggesting that *P. vivax* parasites may have established stable transmission at low levels in sub-Saharan Africa.[15] As only a subset of DN erythroblasts expresses DARC, the scarcity of permissive cells and their restriction to sites of active erythropoiesis may explain why vivax malaria in African DN individuals is often characterized by very low parasitemia.[15–18] Importantly, these data suggest that the extent of the prevalence of *P. vivax* infection in DN African population is currently unknown, as almost all evidence of *P. vivax* infection comes from the conventional detection of parasites in Giemsa-stained blood smears from peripheral blood. The hypothesis that *P. vivax* infections are more widespread than can be deduced from peripheral parasitemia is supported by serological surveys that have shown that *P. vivax* seropositivity in sub-Saharan Africa ranges from 13% to 53% (reviewed in Baird[15]). Diagnosis of vivax malaria based on the microscopic examination of Giemsa-stained blood smears, rapid diagnostic tests, or even NAATs has probably missed many instances of vivax malaria cases, especially in sub-Saharan Africa where DN Africans predominate and in which tropism away from peripheral blood may be more pronounced. Therefore, there is a need for novel diagnostic tools capable of detecting deeper infections to update the current landscape of vivax malaria distribution, frequency, and assessment of clinical impact in sub-Saharan Africa.

On a more positive note, our findings also have important implications for the development of therapeutic approaches and vaccines against *P. vivax* malaria. Indeed, evidence that *P. vivax* merozoites invade DARC+ DN erythroblasts suggests that the leading vaccine candidate targeting the *Pv*DBPII-binding domain of *Pv*DBP in *P. vivax* merozoites[44–46] could be used to prevent vivax malaria in both DP and DN populations.

Although our experimental work represents a breakthrough in our understanding of *P. vivax* invasion pathways, it has several limitations. First, the high inter-individual variability observed has prevented us from fully characterizing DN erythroblasts *in vitro*. Second, the invasion of erythroblasts by *P. vivax* merozoites, in our hands, resulted in few infected cells, probably suggesting that the successful invasion of DN erythroblasts by *P. vivax* merozoites is rare. This low number of infected cells prevents us from quantifying and evaluating the inhibition of *P. vivax* invasion into erythroblasts by anti-DARC antibodies to exclude the potential existence of alternative invasion.[40,41,47,48] An alternative approach could be to perform *in vitro* assays using Duffy-null erythroblasts from immortalized erythroid cell lines (*ackr1* knockout). As the invasion of erythroblasts by *P. vivax* merozoites is highly dependent on culture conditions such as

Figure 3. A subset of genotypically Duffy-negative (DN) erythroblasts expresses DARC during terminal erythroid differentiation (western immunoblotting and immunofluorescence microscopy data)

Figure360▷ For a Figure360 author presentation of this figure, see https://doi.org/10.1016/j.chom.2023.11.007.

(A) Representative western blot analysis of cytoplasmic protein extracts of erythroid cells from one DP (*DP007*) and one DN (*DN005*) donor at days 0, 2, 4, 6, 8, 10, and 12 of terminal differentiation and in erythrocytes from peripheral blood for the presence of DARC and actin (control). Bar graphs represent the relative quantification density of the DARC protein levels for DP007 donor (blue) and DN005 (red), compared with the actin control in each condition.

(B) Additional western blot analyses of cytoplasmic protein extracts of erythroid cells from three DP (*DP001*, *DP003*, and *DP004*) and three DN (*DN001*, *DN003*, and *DN002*) donors at days 0, 10, and 12 (*DP001*/*DN001*), and at days 10 and 12 (*DP003*/*DN003* and *DP004*/*DN002*). Bar graph represents the fold-change in the relative quantification density (and the standard deviation of the mean) of the DARC protein levels for DP donors (*DP001*, *DP003*, and *DP004*, in blue, used as the reference set at 1) and DN donors (*DN001*, *DN002*, and *DN003*, in red), compared with the actin control in each condition. The data were obtained from cytoplasmic protein extracts of erythroid cells collected at days 10 and 12. Error bars show the standard deviation. The relative amount of DARC protein was significantly lower in DN erythroblasts at days 10 and 12 of terminal differentiation compared with DP erythroblasts ($p = 0.03$, Mann-Whitney test).

(C) Fluorescence microscopy of paraformaldehyde-fixed DP (*DP007* and *DP010*) and DN (*DN005* and *DN006*) cells on day 9 of terminal differentiation. Bright-field images are shown on the left, and images with DNA (stained with DAPI, blue) and DARC (stained with anti-DARC antibody, orange) are shown on the right. Objective: 63×.

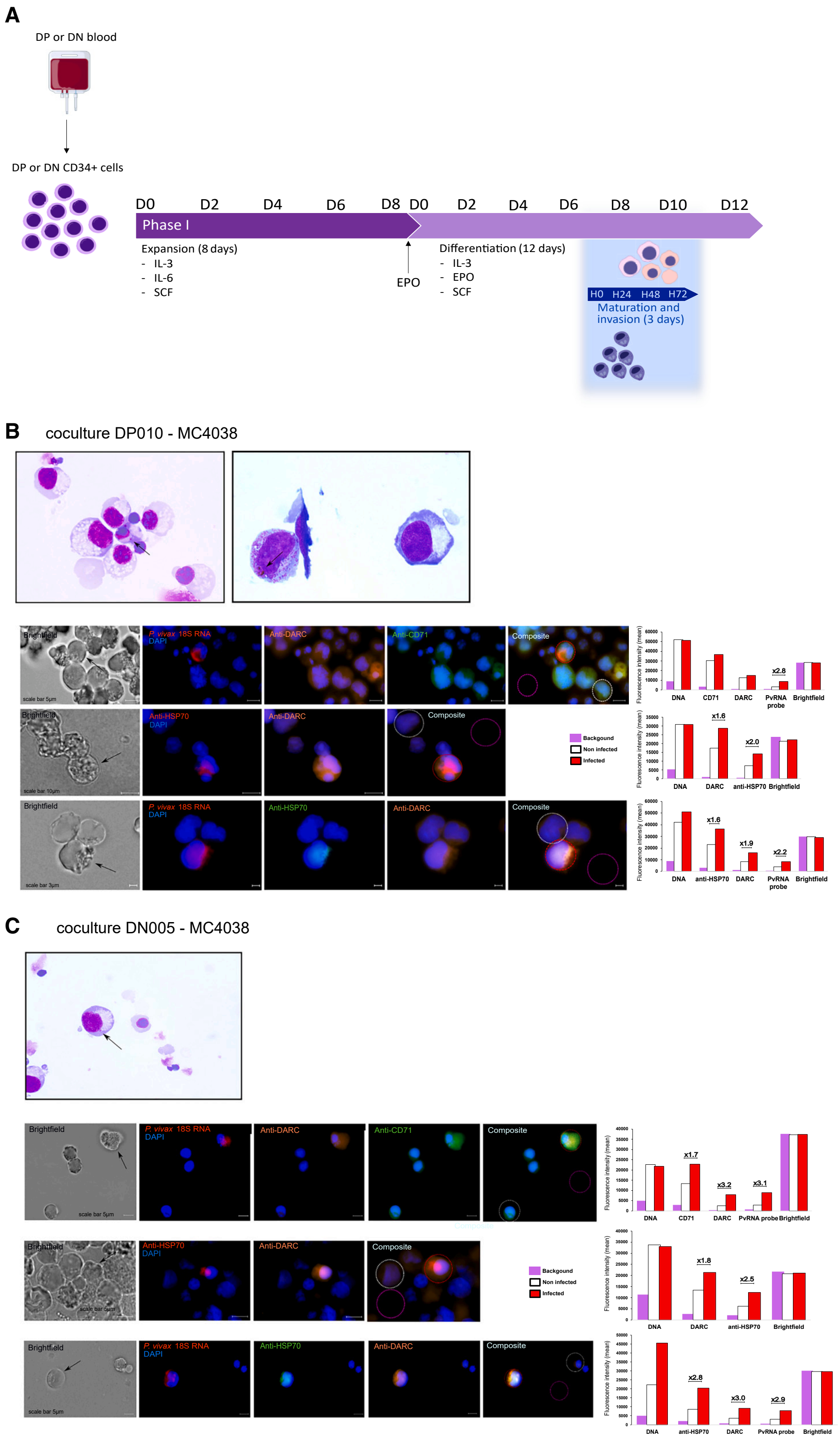

(figure continued on next page)

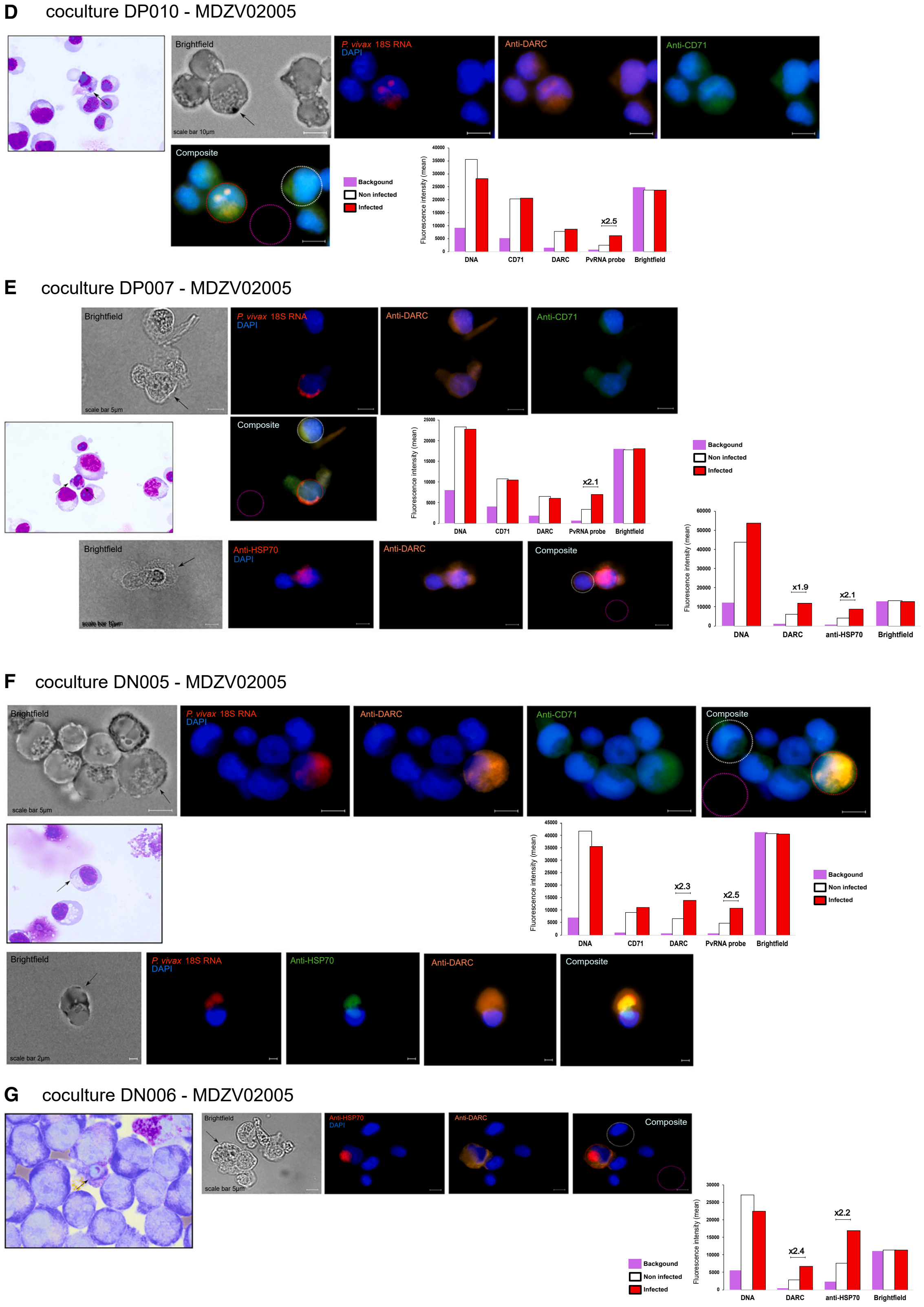

(legend on next page)

the viability of *P. vivax* parasites from cryopreserved parasite samples and parasitemia, performing invasion assays with the use of fresh clinical *P. vivax* isolates from *ex vivo* field studies could also overcome these technical problems. Third, we do not have complete evidence that *in vitro* invasion assays fully replicate the *in vivo* invasion mechanism of *P. vivax*.

Nevertheless, we provide here insights into how genotypically DN erythroblasts can be infected with *P. vivax* merozoites. This finding reveals that a large number of DN individuals may represent a silent reservoir of deep *P. vivax* infections at the sites of active erythropoiesis with very low or no parasitemia and an underestimated biological problem with potential clinical consequences in sub-Saharan Africa.

STAR★METHODS

Detailed methods are provided in the online version of this paper and include the following:

- KEY RESOURCES TABLE
- RESOURCE AVAILABILITY
  - Lead contact
  - Materials availability
  - Data and code availability
- EXPERIMENTAL MODEL AND STUDY PARTICIPANT DETAILS
  - Biological samples
- METHOD DETAILS
  - Duffy genotyping
  - Isolation of human CD34+ cells
  - *In vitro* erythropoiesis assays
  - *In vitro* invasion assays
  - May-Grünwald Giemsa staining and microscopy
  - Antibodies and fluorescent dyes
  - Flow cytometry
  - Protein extraction and western immunoblot
  - Fluorescence microscopy
  - Statistical analysis

SUPPLEMENTAL INFORMATION

Supplemental information can be found online at https://doi.org/10.1016/j.chom.2023.11.007.

ACKNOWLEDGMENTS

We would like to thank all the patients and donors who agreed to provide blood samples, Sabine Haas (Etablissement Français du Sang de Strasbourg) for coordinating the blood donations, and Olivier Silvie (Centre d'Immunologie et des Maladies Infectieuses de Paris) for providing anti-HSP70 polyclonal antibody. This study was supported by a grant overseen by the French National Research Agency (VIPeRs, ANR-18-CE15-0026), the Institut Pasteur, Paris, the Laboratoire d'Excellence (LabEx) "French Parasitology Alliance for Health Care" (ANR-11-15 LABX-0024-PARAFRAP), and the University of Strasbourg through the Programme IdEX 2022 (Attractivité, Dotation d'accueil des professeurs d'université recrutés en 2021). *P. vivax* samples collection in Ethiopia was supported by a grant from the Bill and Melinda Gates Foundation (ID: INV-015996).

I.B. has received financial support from the French National Research Agency (VIPeRs, ANR-18-CE15-0026), the Fondation pour la Recherche Médicale (grant agreement number FRM FDT202204014872), the European Hematology Association (grant agreement number RMG-202211-02941), and the Fondation Thérèse Lebrasseur (D.M. was awarded the Thérèse Lebrasseur Foundation Prize in 2021). S.E.H. received funding from the European Union's Horizon 2020 Research and Innovation Programme under the Marie Sklodowska-Curie grant agreement number 101024970.

AUTHOR CONTRIBUTIONS

I.B., S.E.H., J.S., and D.M. designed the study; M.S., L.T.R., I.V.-W., A.R., A.A., and L.G. provided the biological samples; I.B., C.D.-L., S.M., M.G.-B., F.J.M., S.E.H., J.S., and D.M. conducted and supervised the experiments; I.B., P.K., and D.M. acquired the data; I.B., C.D.-L., C.E.C., S.E.H., J.S., P.K., and D.M. analyzed the data; I.B., C.E.C., S.E.H., J.S., and D.M. wrote the manuscript. All authors have read and edited the manuscript.

DECLARATION OF INTERESTS

The authors declare no competing interests.

Figure 4. *P. vivax* merozoites can invade DN erythroblasts during terminal erythroid differentiation

Figure360▷ For a Figure360 author presentation of this figure, see https://doi.org/10.1016/j.chom.2023.11.007.

(A) The diagram illustrates the two-phase *in vitro* culture system for the isolation of CD34+ cells, the expansion and differentiation of erythroid progenitors, the maturation of *P. vivax* cryopreserved isolates and *in vitro* invasion assays. The details are provided in the STAR Methods section.

(B–G) *P. vivax* parasites (black arrows) were observed after 24 h of co-culture on May Grunwald-Giemsa-stained slides by light microscopy and fluorescence microscopy (bright field + DAPI [blue]/RNA 18S FISH probe [red] + anti-DARC antibody [orange] + anti-CD71 antibody [green] or bright field + DAPI [blue]/anti-HSP70 antibody [red] + anti-DARC antibody [orange] or bright field + DAPI [blue]/RNA 18S FISH probe [red] + anti-HSP70 antibody [green] + anti-DARC antibody [orange]; objective: 63×). The bar graphs represent the mean fluorescence intensity (MFI) of the background (purple), non-infected cells (white), and *P. vivax*-infected cells (red) detected by fluorescence microscopy (DAPI [DNA, blue], RNA 18S FISH probe [red], anti-HSP70 antibody [red or green], anti-DARC antibody [orange], and anti-CD71 antibody green]). A scale bar is shown on each image. Additional details are provided in the method details section and Table S1.

(B) Co-culture DP010-MC4038. DP erythroblasts (at day 9 of terminal differentiation) infected with an Ethiopian *P. vivax* isolate were observed by light microscopy (ring stage in the top left panel and mature stage with hemozoin in the top right panel) and fluorescence microscopy (second, third, and fourth line panels).

(C) Co-culture DN005-MC4038. DN erythroblasts (at day 9 of terminal differentiation) infected with the same Ethiopian *P. vivax* isolate observed by light microscopy (ring stage, top panel) and fluorescence microscopy (second, third, and fourth lines panel).

(D) Co-culture DP010-MDZV02005. DP erythroblasts (at day 9 of terminal differentiation) infected with a Malagasy *P. vivax* isolate observed by light microscopy (mature ring stage) and fluorescence microscopy (right panel).

(E) Co-culture DP007-MDZV02005. DP erythroblasts (at day 9 of terminal differentiation) were infected with the same Malagasy *P. vivax* isolate using light microscopy (two ring stages) and fluorescence microscopy.

(F) Co-culture DN005-MDZV02005. DN erythroblasts (at day 9 of terminal differentiation) were infected with the same Malagasy *P. vivax* isolate observed by light microscopy (ring stage) and fluorescence microscopy.

(G) Co-culture DN006-MDZV02005. DN erythroblasts (at day 9 of terminal differentiation) infected with the same Malagasy *P. vivax* isolate observed by light microscopy (mature stage) and fluorescence microscopy.

CellPress
OPEN ACCESS

INCLUSION AND DIVERSITY

We support inclusive, diverse, and equitable conduct of research.

Received: June 6, 2023
Revised: October 1, 2023
Accepted: November 8, 2023
Published: December 5, 2023

REFERENCES

1. World Health Organization (2022). World malaria report 2022. https://www.mmv.org/newsroom/news-resources-search/world-malaria-report-2022?gclid=EAIaIQobChMIsLiygPHKggMVOKtmAh2UrQifEAAYASAAEgLd3vD_BwE.
2. Boyd, M.P., and Stratman-Thomas, W.K. (1933). Studies on benign tertian malaria. I. On the occurrence of acquired tolerance to Plasmodium vivax. Am. J. Epidemiol. *17*, 55–59.
3. Bray, R.S. (1958). The susceptibility of Liberians to the Madagascar strain of Plasmodium vivax. J. Parasitol. *44*, 371–373.
4. Young, M.D., Ellis, J.M., and Stubbs, T.H. (1947). Some characteristics of foreign vivax malaria induced in neurosyphilitic patients. Am. J. Trop. Med. Hyg. *s1–27*, 585–596.
5. Young, M.D., Eyles, D.E., Burgess, R.W., and Jeffery, G.M. (1955). Experimental testing of the immunity of negroes to Plasmodium vivax. J. Parasitol. *41*, 315–318.
6. Cutbush, M., and Mollison, P.L. (1950). The Duffy blood group system. Heredity *4*, 383–389.
7. Cutbush, M., Mollison, P.L., and Parkin, D.M. (1950). A new human blood group. Nature *165*, 188–189.
8. Miller, L.H., Mason, S.J., Clyde, D.F., and McGinniss, M.H. (1976). The resistance factor to Plasmodium vivax in blacks. The Duffy-blood-group genotype, FyFy. N. Engl. J. Med. *295*, 302–304.
9. Barnwell, J.W., Nichols, M.E., and Rubinstein, P. (1989). In vitro evaluation of the role of the Duffy blood group in erythrocyte invasion by Plasmodium vivax. J. Exp. Med. *169*, 1795–1802.
10. Chitnis, C.E., and Miller, L.H. (1994). Identification of the erythrocyte binding domains of Plasmodium vivax and Plasmodium knowlesi proteins involved in erythrocyte invasion. J. Exp. Med. *180*, 497–506.
11. Gruszczyk, J., Kanjee, U., Chan, L.J., Menant, S., Malleret, B., Lim, N.T.Y., Schmidt, C.Q., Mok, Y.F., Lin, K.M., Pearson, R.D., et al. (2018). Transferrin receptor 1 is a reticulocyte-specific receptor for Plasmodium vivax. Science *359*, 48–55.
12. Malleret, B., El Sahili, A., Tay, M.Z., Carissimo, G., Ong, A.S.M., Novera, W., Lin, J., Suwanarusk, R., Kosaisavee, V., Chu, T.T.T., et al. (2021). Plasmodium vivax binds host CD98hc (SLC3A2) to enter immature red blood cells. Nat. Microbiol. *6*, 991–999.
13. Malleret, B., Li, A., Zhang, R., Tan, K.S.W., Suwanarusk, R., Claser, C., Cho, J.S., Koh, E.G.L., Chu, C.S., Pukrittayakamee, S., et al. (2015). Plasmodium vivax: restricted tropism and rapid remodeling of CD71-positive reticulocytes. Blood *125*, 1314–1324.
14. Bouyssou, I., Martínez, F.J., Campagne, P., Ma, L., Doderer-Lang, C., Chitnis, C.E., and Ménard, D. (2022). *Plasmodium vivax* blood stage invasion pathways: contribution of omics technologies in deciphering molecular and cellular mechanisms. C. R. Biol. *345*, 91–133.
15. Baird, J.K. (2022). African Plasmodium vivax malaria improbably rare or benign. Trends Parasitol. *38*, 683–696.
16. Abate, A., Bouyssou, I., Mabilotte, S., Doderer-Lang, C., Dembele, L., Menard, D., and Golassa, L. (2022). Vivax malaria in Duffy-negative patients shows invariably low asexual parasitaemia: implication towards malaria control in Ethiopia. Malar. J. *21*, 230.
17. Albsheer, M.M.A., Pestana, K., Ahmed, S., Elfaki, M., Gamil, E., Ahmed, S.M., Ibrahim, M.E., Musa, A.M., Lo, E., and Hamid, M.M.A. (2019). Distribution of Duffy phenotypes among Plasmodium vivax infections in Sudan. Genes *10*, 437.
18. Lo, E., Russo, G., Pestana, K., Kepple, D., Abagero, B.R., Dongho, G.B.D., Gunalan, K., Miller, L.H., Hamid, M.M.A., Yewhalaw, D., et al. (2021). Contrasting epidemiology and genetic variation of Plasmodium vivax infecting Duffy-negative individuals across Africa. Int. J. Infect. Dis. *108*, 63–71.
19. Barber, B.E., William, T., Grigg, M.J., Parameswaran, U., Piera, K.A., Price, R.N., Yeo, T.W., and Anstey, N.M. (2015). Parasite biomass-related inflammation, endothelial activation, microvascular dysfunction and disease severity in vivax malaria. PLoS Pathog. *11*, e1004558.
20. Brito, M.A.M., Baro, B., Raiol, T.C., Ayllon-Hermida, A., Safe, I.P., Deroost, K., Figueiredo, E.F.G., Costa, A.G., Armengol, M.D.P., Sumoy, L., et al. (2022). Morphological and transcriptional changes in human bone marrow during natural Plasmodium vivax malaria infections. J. Infect. Dis. *225*, 1274–1283.
21. Fonseca, L.L., Joyner, C.J., Saney, C.L., MaHPIC Consortium, Moreno, A., Barnwell, J.W., Galinski, M.R., and Voit, E.O. (2018). Analysis of erythrocyte dynamics in rhesus macaque monkeys during infection with Plasmodium cynomolgi. Malar. J. *17*, 410.
22. Lacerda, M.V., Hipólito, J.R., and Passos, L.N. (2008). Chronic Plasmodium vivax infection in a patient with splenomegaly and severe thrombocytopenia. Rev. Soc. Bras. Med. Trop. *41*, 522–523.
23. Obaldia, N., 3rd, Meibalan, E., Sa, J.M., Ma, S., Clark, M.A., Mejia, P., Moraes Barros, R.R., Otero, W., Ferreira, M.U., Mitchell, J.R., et al. (2018). Bone marrow is a major parasite reservoir in Plasmodium vivax infection. mBio *9*, e00625-18.
24. O'Donnell, J., Goldman, J.M., Wagner, K., Ehinger, G., Martin, N., Leahy, M., Kariuki, N., Dokal, I., and Roberts, I. (1998). Donor-derived Plasmodium vivax infection following volunteer unrelated bone marrow transplantation. Bone Marrow Transplant. *21*, 313–314.
25. Ru, Y.X., Mao, B.Y., Zhang, F.K., Pang, T.X., Zhao, S.X., Liu, J.H., and Wickramasinghe, S.N. (2009). Invasion of erythroblasts by Pasmodium vivax: a new mechanism contributing to malarial anemia. Ultrastruct. Pathol. *33*, 236–242.
26. Shaikh, M.S., Ali, B., Janjua, M., Akbar, A., Haider, S.A., Moiz, B., Raheem, A., Baird, J.K., and Beg, M.A. (2021). Plasmodium in the bone marrow: case series from a hospital in Pakistan, 2007-2015. Malar. J. *20*, 254.
27. Silva-Filho, J.L., Dos-Santos, J.C., Judice, C., Beraldi, D., Venugopal, K., Lima, D., Nakaya, H.I., De Paula, E.V., Lopes, S.C., Lacerda, M.V., et al. (2021). Total parasite biomass but not peripheral parasitaemia is associated with endothelial and haematological perturbations in Plasmodium vivax patients. eLife *10*, e71351.
28. del Portillo, H.A., Lanzer, M., Rodriguez-Malaga, S., Zavala, F., and Fernandez-Becerra, C. (2004). Variant genes and the spleen in Plasmodium vivax malaria. Int. J. Parasitol. *34*, 1547–1554.
29. Fernandez-Becerra, C., Bernabeu, M., Castellanos, A., Correa, B.R., Obadia, T., Ramirez, M., Rui, E., Hentzschel, F., López-Montañés, M., Ayllon-Hermida, A., et al. (2020). Plasmodium vivax spleen-dependent genes encode antigens associated with cytoadhesion and clinical protection. Proc. Natl. Acad. Sci. USA *117*, 13056–13065.
30. Kho, S., Qotrunnada, L., Leonardo, L., Andries, B., Wardani, P.A.I., Fricot, A., Henry, B., Hardy, D., Margyaningsih, N.I., Apriyanti, D., et al. (2021). Evaluation of splenic accumulation and colocalization of immature reticulocytes and Plasmodium vivax in asymptomatic malaria: a prospective human splenectomy study. PLoS Med. *18*, e1003632.
31. Mende, N., and Laurenti, E. (2021). Hematopoietic stem and progenitor cells outside the bone marrow: where, when, and why. Exp. Hematol. *104*, 9–16.
32. Hu, J., Liu, J., Xue, F., Halverson, G., Reid, M., Guo, A., Chen, L., Raza, A., Galili, N., Jaffray, J., et al. (2013). Isolation and functional characterization of human erythroblasts at distinct stages: implications for understanding of normal and disordered erythropoiesis in vivo. Blood *121*, 3246–3253.
33. Tournamille, C., Colin, Y., Cartron, J.P., and Le Van Kim, C. (1995). Disruption of a GATA motif in the Duffy gene promoter abolishes erythroid gene expression in Duffy–negative individuals. Nat. Genet. *10*, 224–228.

34. Southcott, M.J.G., Tanner, M.J.A., and Anstee, D.J. (1999). The expression of human blood group antigens during erythropoiesis in a cell culture system. Blood *93*, 4425–4435.
35. Moras, M., Lefevre, S.D., and Ostuni, M.A. (2017). From erythroblasts to mature red blood cells: organelle clearance in mammals. Front. Physiol. *8*, 1076.
36. Dong, H.Y., Wilkes, S., and Yang, H. (2011). CD71 is selectively and ubiquitously expressed at high levels in erythroid precursors of all maturation stages: a comparative immunochemical study with glycophorin A and hemoglobin A. Am. J. Surg. Pathol. *35*, 723–732.
37. Brun, A., Gaudernack, G., and Sandberg, S. (1990). A new method for isolation of reticulocytes: positive selection of human reticulocytes by immunomagnetic separation. Blood *76*, 2397–2403.
38. Kono, M., Kondo, T., Takagi, Y., Wada, A., and Fujimoto, K. (2009). Morphological definition of CD71 positive reticulocytes by various staining techniques and electron microscopy compared to reticulocytes detected by an automated hematology analyzer. Clin. Chim. Acta *404*, 105–110.
39. Malleret, B., Xu, F., Mohandas, N., Suwanarusk, R., Chu, C., Leite, J.A., Low, K., Turner, C., Sriprawat, K., Zhang, R., et al. (2013). Significant biochemical, biophysical and metabolic diversity in circulating human cord blood reticulocytes. PLoS One *8*, e76062.
40. Kanjee, U., Grüring, C., Babar, P., Meyers, A., Dash, R., Pereira, L., Mascarenhas, A., Chaand, M., Rangel, G.W., Clark, M.A., et al. (2021). Plasmodium vivax Strains use alternative pathways for invasion. J. Infect. Dis. *223*, 1817–1821.
41. Ntumngia, F.B., Thomson-Luque, R., Torres, L.d.M., Gunalan, K., Carvalho, L.H., and Adams, J.H. (2016). A novel erythrocyte binding protein of Plasmodium vivax suggests an alternate invasion pathway into Duffy-positive reticulocytes. mBio *7*.
42. Oboh-Imafidon, M.A., and Zimmerman, P.A. (2023). Plasmodium vivax in Sub-Saharan Africa: an advancing threat to malaria elimination? Am. J. Trop. Med. Hyg. *109*, 497–498.
43. Quaye, I.K., Aleksenko, L., Paganotti, G.M., Peloewetse, E., Haiyambo, D.H., Ntebela, D., Oeuvray, C., and Greco, B.; The Pavon Consortium (2023). Malaria elimination in Africa: rethinking strategies for Plasmodium vivax and lessons from Botswana. Trop Med Infect Dis *8*, 392.
44. Hou, M.M., Barrett, J.R., Themistocleous, Y., Rawlinson, T.A., Diouf, A., Martinez, F.J., Nielsen, C.M., Lias, A.M., King, L.D.W., Edwards, N.J., et al. (2022). Impact of a blood-stage vaccine on Plasmodium vivax malaria. Preprint at medRxiv.
45. Payne, R.O., Silk, S.E., Elias, S.C., Milne, K.H., Rawlinson, T.A., Llewellyn, D., Shakri, A.R., Jin, J., Labbé, G.M., Edwards, N.J., et al. (2017). Human vaccination against *Plasmodium vivax* Duffy-binding protein induces strain-transcending antibodies. JCI Insight *2*, e93683.
46. Singh, K., Mukherjee, P., Shakri, A.R., Singh, A., Pandey, G., Bakshi, M., Uppal, G., Jena, R., Rawat, A., Kumar, P., et al. (2018). Malaria vaccine candidate based on Duffy-binding protein elicits strain transcending functional antibodies in a phase I trial. npj Vaccines *3*, 48.
47. Gunalan, K., Sá, J.M., Moraes Barros, R.R., Anzick, S.L., Caleon, R.L., Mershon, J.P., Kanakabandi, K., Paneru, M., Virtaneva, K., Martens, C., et al. (2019). Transcriptome profiling of Plasmodium vivax in Saimiri monkeys identifies potential ligands for invasion. Proc. Natl. Acad. Sci. USA *116*, 7053–7061.
48. Lee, S.K., Low, L.M., Andersen, J.F., Yeoh, L.M., Valenzuela Leon, P.C., Drew, D.R., Doehl, J.S.P., Calvo, E., Miller, L.H., Beeson, J.G., et al. (2023). The direct binding of Plasmodium vivax AMA1 to erythrocytes defines a RON2-independent invasion pathway. Proc. Natl. Acad. Sci. USA *120*, e2215003120.
49. Ménard, D., Barnadas, C., Bouchier, C., Henry-Halldin, C., Gray, L.R., Ratsimbasoa, A., Thonier, V., Carod, J.F., Domarle, O., Colin, Y., et al. (2010). Plasmodium vivax clinical malaria is commonly observed in Duffy-negative Malagasy people. Proc. Natl. Acad. Sci. USA *107*, 5967–5971.
50. El Hoss, S., Cochet, S., Godard, A., Yan, H., Dussiot, M., Frati, G., Boutonnat-Faucher, B., Laurance, S., Renaud, O., Joseph, L., et al. (2021). Fetal hemoglobin rescues ineffective erythropoiesis in sickle cell disease. Haematologica *106*, 2707–2719.
51. El Hoss, S., Cochet, S., Marin, M., Lapouméroulie, C., Dussiot, M., Bouazza, N., Elie, C., de Montalembert, M., Arnaud, C., Guitton, C., et al. (2019). Insights into determinants of spleen injury in sickle cell anemia. Blood Adv. *3*, 2328–2336.

STAR★METHODS

KEY RESOURCES TABLE

REAGENT or RESOURCE	SOURCE	IDENTIFIER
Antibodies		
BV421-conjugated mouse monoclonal anti-CD235a (GPA) antibody (flow cytometry)	BD Biosciences	Cat.# 562938 (clone GA-R2); RRID: AB_2721016
BV421-conjugated mouse monoclonal anti-mouse CD71 antibody (flow cytometry)	BD Biosciences	Cat.# 562995 (clone M-A712); RRID: AB_2737939
PECy7-conjugated mouse monoclonal anti-CD235a (GPA) antibody (flow cytometry)	BD Pharmingen	Cat.# 563666 (clone GA-R2); RRID: AB_2738361
APC-conjugated mouse monoclonal anti-CD36 antibody (flow cytometry)	BD Pharmingen	Cat.# 562744 (clone CRF D-2712); RRID: AB_2737763
APC-conjugated mouse monoclonal anti-CD49d antibody (flow cytometry)	BD Pharmingen	Cat.# 559881 (clone 9F10)
PE-conjugated mouse monoclonal anti-DARC antibody (flow cytometry)	BD Pharmingen	Cat.# 566424 (clone NaM185-2C3); RRID: AB_2739724
FITC-conjugated mouse monoclonal anti-Band 3 antibody (flow cytometry)	IBGRL	Cat.# 9439FITC (clone BRIC6)
APC-conjugated recombinant human monoclonal anti-DARC antibody (flow cytometry)	Miltenyi Biotec	Cat.# 130-120-239 (clone REA376)
FITC-conjugated mouse monoclonal anti-CD71 antibody (flow cytometry)	Miltenyi Biotec	Cat.# 130-126-032 (clone AC102); RRID: AB_615104
Rabbit polyclonal anti-DARC antibody (immunoblotting)	Sigma-Aldrich	Cat.# SAB3500242
Goat anti-rabbit IgG monoclonal antibody (immunoblotting)	Cell Signaling Technology	Cat.# 7074; RRID: AB_2099233
Rabbit anti-DARC monoclonal antibody (fluorescence)	Thermo Fisher Scientific	Cat.# 703703 (clone 10H52L38); RRID: AB_2809247
Mouse anti-CD71 monoclonal antibody (fluorescence)	Thermo Fisher Scientific	Cat.# 14-0719-82 (clone OKT9); RRID: AB_467338
Mouse anti-HSP70 polyclonal antibody (fluorescence)	Pr. Olivier Silvie, Biology and Immunology of Malaria Research Unit, CIMI	N/A
Alexa Fluor 555-conjugated anti-rabbit IgG goat polyclonal antibody (fluorescence)	Invitrogen	Cat.# A-21429; RRID: AB_2535850
Alexa Fluor 488-conjugated anti-mouse IgG goat polyclonal antibody (fluorescence)	Invitrogen	Cat.# A-28175; RRID: AB_2536161
Biological samples		
Human CD34+ cells	This study	Cf. Tables 1 and S1
P. vivax isolates (Madagascar)	Matthieu Schoenhals<br>Demande d'accès aux ressources génétiques No. 164/2020/DIR/HM (10/07/2020)	Cf. Table S1
P. vivax isolates (Ethiopia)	Lemu Golassa<br>Approval od shipment of sampels (No. MoSHE/04/246/967/21)	Cf. Table S1
Cellular stains		
7-AAD fluorochrome (flow cytometry)	BD Pharmingen	Cat.# 559925
FITC-conjugated Annexin V dye (flow cytometry)	BD Pharmingen	Cat.# 556419
Hoechst 33342 dye (flow cytometry)	BD Pharmingen	Cat.# 561908

(*Continued on next page*)

REAGENT or RESOURCE	SOURCE	IDENTIFIER
Continued		
Oligonucleotides		
Quasar-670-conjugated RNA Stellaris FISH probe	Biosearch Technologies	www.biosearchtech.com/stellarisdesigner
Software and algorithms		
FlowJo v.10	BD Biosciences	https://www.flowjo.com/solutions/flowjo/downloads
Diva v. 8	BD Biosciences	https://www.bdbiosciences.com/en-us/products/software/instrument-software/bd-facsdiva-software
Image Lab v.6.1	Bio-rad	https://www.bio-rad.com/fr-fr/product/image-lab-software
GraphPad Prism	GraphPad	https://www.graphpad.com/features
Zen v.3.7	Carl Zeiss	https://www.zeiss.com/microscopy/fr/produits/logiciel/zeiss-zen
Instruments		
Flow cytometer LSR Fortessa	BD Biosciences	https://www.bdbiosciences.com/en-fr/products/instruments/flow-cytometers/research-cell-analyzers/bd-lsrfortessa
Flow cytometer Attune NxT	Thermo Fisher Scientific	https://www.thermofisher.com/fr/fr/home/life-science/cell-analysis/flow-cytometry/flow-cytometers/attune-nxt-flow-cytometer/models/nxt.html
Apotome microscope	Carl Zeiss	https://www.zeiss.com/microscopy/fr/produits/microscopes-optiques/microscopes-a-champ-large.html
Axio Imager M2 upright microscope	Carl Zeiss	https://www.zeiss.com/microscopy/fr/produits/microscopes-optiques/microscopes-a-champ-large.html
ChemiDoc Touch Imaging System	Biorad	https://www.bio-rad.com/en-uk/product/chemidoc-touch-imaging-system?ID=NINJCT4VY
REBEL ECHO Microscope	ECHO	https://discover-echo.com/rebel/?utm_source=google&utm_medium=search&utm_campaign=11514218595&utm_term=echo%20rebel%20microscope&utm_content=476427864927&gad_source=1&gclid=CjwKCAiAjfyqBhAsEiwA-UdzJEiSGIUayptitA5jp_437ITDDyrkOidILmCHnVFzMxPVBZpmuhXZohoCd7oQAvD_BwE

RESOURCE AVAILABILITY

Lead contact
Further information and requests for resources and reagents should be directed to and will be fulfilled by the lead contact, Didier Menard (dmenard@pasteur.fr).

Materials availability
No new reagents were generated in this study.

Data and code availability
Original western blot images and microscopy data reported in this paper could be shared by the lead contact upon request. This paper does not report original code. Any additional information required to reanalyze the data reported in this paper is available from the lead contact upon request.

EXPERIMENTAL MODEL AND STUDY PARTICIPANT DETAILS

Biological samples

Whole blood from healthy donors

Whole blood samples from Duffy-positive (DP) and Duffy-negative (DN) healthy donors were provided by the Etablissement Français du Sang (EFS) in Strasbourg, according to the Ethics Committees of the EFS and the University of Strasbourg. All patients provided informed consent, and when the participants were children, their parents or guardians provided consent.

P. vivax *clinical isolates*

Clinical *P. vivax* isolates were obtained from symptomatic patients infected with *P. vivax* and collected in health centers in Madagascar according to the Declaration of Helsinki, with approval from the Malagasy Ministry of Health, National Ethics Committee (N°050 MSANP/SG/-AGMED/CNPV/CERBM) and the Institut Pasteur Department of Legal Affairs (IRB/2019/04). All patients provided informed consent, and when the participants were children, their parents or guardians provided consent. Whole blood samples were cryopreserved in liquid nitrogen (LN). *P. vivax* infected patients were treated with antimalarial drugs according to the national treatment guidelines based on their microscopic result.

In Ethiopia, clinical *P. vivax* isolates were obtained from symptomatic patients visiting the Adama Malaria Diagnostic Center after confirmation with thick and thin blood films. Approximately 2 ml of venous blood was collected in EDTA tubes from each study participant and shipped to the University of Strasbourg (Strasbourg, France). Ethical approval was obtained from the Institutional Review Board of Aklilu Lemma Institute of Pathobiology (No. ALIPB IRB/18/2012/20) and National Research Ethics Review Committee (NRERC) (No. MoSHE/02/152/778/21). The study participants, their parents, and/or guardians were informed about the purpose, procedure, and benefit of the study and the minor side effects of the procedure during blood sample collection. Blood samples were obtained after obtaining written informed consent and/or assent from all study participants or their parents/guardians/caregivers. *P. vivax* infected patients were treated with antimalarial drugs according to the national treatment guideline based on their microscopic result.

METHOD DETAILS

Duffy genotyping

The Duffy genotype of the blood donors was determined using Sanger sequencing. Briefly, genomic DNA was extracted from blood samples using a QiaAmp DNA kit (Qiagen), according to the supplier's recommendations. Duffy gene amplification was performed by nested polymerase chain reaction (PCR) to search for potential GATA-1 and Fy mutations. The PCR products were sequenced using Sanger sequencing, as previously described.[49]

Isolation of human CD34+ cells

Peripheral blood mononuclear cells (PBMCs) from Duffy-positive (DP) and Duffy-negative (DN) donors were isolated from whole blood after Ficoll fractionation (G&E Healthcare). CD34+ cells were isolated by positive magnetic sorting using a CD34+ progenitor cell isolation kit (Miltenyi Biotec), according to the supplier's protocol. CD34+ cells were then frozen in freezing medium containing 10% dimethyl sulfoxide (DMSO) in liquid nitrogen and used for downstream analysis.[50]

In vitro erythropoiesis assays

Duffy-positive (DP) and Duffy-negative (DN) erythroid progenitors were placed in a two-phase *in vitro* culture system for the differentiation of human erythroid progenitors.[50] Briefly, CD34+ cells were expanded in Iscove's Modified Dulbecco's Medium (IMDM, Gibco) supplemented with 15% BIT9500 medium (Stem Cell Technologies), 100 U/ml Penicillin Streptomycin (Gibco), 2mM L-Glutamine (Gibco), 10ng/ml ng/mL human recombinant interleukin-3 (IL-3), 100ng/ml ng/mL human recombinant interleukin-6 (IL-6) and 50ng/ml human recombinant stem cell factor (SCF) (Miltenyi Biotec). This expansion phase lasted for 8 days. The cells were maintained at 2×10^5 cells/ml, and the medium was changed every other day. Differentiation was initiated using IMDM (Gibco) supplemented with 15% BIT9500 medium (Stem Cell Technologies), 100 U/ml Penicillin Streptomycin (Gibco), 2mM L-Glutamine (Gibco), 10ng/ml ng/mL human IL-3, 2U/ml U/mL erythropoietin (EPO) and 50ng/ml human SCF (Miltenyi Biotech). The differentiation phase lasted 12 days. Cells were maintained at 2×10^5 cells/ml until day 8 and, from then onward, at 5×10^5 cells/ml with the medium changed every other day.

In vitro invasion assays

Duffy-positive (DP) and Duffy-negative (DN) erythroid progenitors were placed in a two-phase (expansion and differentiation) *in vitro* culture system.[50] At Day 7 of terminal differentiation, *P. vivax* cryoisolates were thawed, placed in IMDM (Gibco) supplemented with 10% AB+ human serum (Sigma Aldrich), 10mM hypoxanthine (Dutcher) and 50µg/ml gentamycin (Fisher Scientific) and incubated at 37°C under gas conditions (5% O_2, 5% CO_2, 90% N_2) for approximately 30h. At Day 8 of terminal differentiation, *P. vivax* parasites were placed in co-culture with either Duffy-positive (DP) or Duffy-negative (DN) erythroblasts and incubated at 37°C under gas conditions (5% O_2, 5% CO_2, 90% N_2) for 48h.

May-Grünwald Giemsa staining and microscopy

After 24 and 48 hours of co-culture, erythroblasts were harvested, washed, and spun on glass slides at 3000rpm for 3 min using a Cytospin centrifuge (Shandon). The dot smears were stained with May-Grünwald Giemsa (MGG) staining (Sigma-Aldrich) following the manufacturer's instructions, and then observed by optical microscopy (Objective 100X) (EchoRebel).

Antibodies and fluorescent dyes

The BV421-conjugated anti-glycophorin A (GPA) mouse monoclonal antibody, PECy7-conjugated anti-GPA mouse monoclonal antibody, BV421-conjugated anti-CD71 mouse monoclonal antibody, APC-conjugated anti-CD36 mouse monoclonal antibody (BD Biosciences), APC-conjugated anti-CD49d mouse monoclonal antibody, PE-conjugated anti-DARC rabbit monoclonal antibody (BD Pharmingen), FITC-conjugated anti-band 3 mouse monoclonal antibody (IBGRL), APC-conjugated anti-DARC rabbit monoclonal antibody, APC-conjugated anti-GPA recombinant human monoclonal antibody, FITC-conjugated anti-CD71 mouse monoclonal antibody (Miltenyi biotec), 7-Aminoactinomycin D (7AAD) fluorochrome, FITC-conjugated annexin V dye (BD Biosciences) and Hoechst 33342 dye (Invitrogen) were used for the follow up of the characterization of Duffy-negative erythroblasts and invasion assays by flow cytometry.

Anti-DARC rabbit polyclonal antibody (Sigma-Aldrich) and anti-rabbit IgG goat monoclonal antibody (Cell Signaling Technology) were used to detect DARC by western blotting.

The anti-DARC rabbit monoclonal antibody, anti-CD71 mouse monoclonal antibody (Fisher), Alexa Fluor 555-conjugated anti-rabbit IgG goat polyclonal antibody, Alexa Fluor 488-conjugated anti-mouse IgG goat polyclonal antibody (Invitrogen), and a set of RNA Stellaris FISH probes specific to *P. vivax* 18S RNA and conjugated to quasar-670 (Stellaris) were used for fluorescence microscopy. The sequences of the probes were designed with the Stellaris® FISH probe designer (Biosearch technologies) available online at www.biosearchtech.com/stellarisdesigner.

Flow cytometry

Erythroid differentiation staining assay

Cells were harvested and stained for surface expression of GPA, Band 3, and CD49d on D0, D2, D4, D6, D8, D10, and D12 of terminal differentiation. Specifically, 1×10^5 cells were washed with PBS (1X) + 0.2% BSA and incubated with fluorescence-conjugated antibodies at room temperature for 30 min in the dark. The cells were washed twice with PBS (1X) + 0.2% BSA and incubated with 7-AAD for 5 min prior to analysis. Samples were analyzed using an LSR Fortessa flow cytometer and acquired using the Diva software version 8 (BD Biosciences). The data were analyzed using FlowJo software version 10 (BD Biosciences).

Duffy staining assay

Cells were harvested and stained for surface expression of GPA, DARC and CD71 at D0, D2, D4, D6, D8, D10 and D12 of terminal differentiation. Specifically, 1×10^5 cells were washed with PBS (1X) + 0.2% BSA and incubated with fluorescence-conjugated antibodies at room temperature for 30 min in the dark. They were then washed twice with PBS (1X) + 0.2% BSA. The cells were analyzed using an LSR Fortessa flow cytometer (BD Biosciences) and an Attune NxT Acoustic Focusing flow cytometer (Thermo Fischer Scientific). Acquisition was performed using the Diva software (BD Biosciences) and Attune NxT software (Thermo Fischer Scientific). The data were analyzed using FlowJo software v.10 (BD Biosciences).

Apoptosis staining assay

Cells were harvested and stained to measure the percentage of apoptotic cells on D0, D2, D4, D6, D8, D10, and D12 of terminal differentiation. Specifically, 1×10^5 cells were washed once with annexin buffer and incubated with FITC-conjugated Annexin V dye (BD Biosciences) at room temperature for 20 min in the dark. They were analyzed using an LSR Fortessa flow cytometer and acquired using the Diva software version 8 (BD Biosciences). The data were analyzed using FlowJo software version 10 (BD Biosciences).

Enucleation staining assay

Cells were harvested and stained to measure the percentage of enucleated cells on D8, D10, and D12 of terminal differentiation. Specifically, 1×10^5 cells were washed with PBS (1X) + 0.2% BSA and incubated with PECy7-conjugated anti-GPA and APC-conjugated anti-CD36 mouse monoclonal antibodies (BD Biosciences) at room temperature for 30 min in the dark. The cells were washed twice with PBS (1X) + 0.2% BSA and incubated with Hoechst 33342 dye (Invitrogen) for 60 min at 37°C. They were analyzed using an LSR Fortessa flow cytometer and acquired using the Diva software version 8 (BD Biosciences). The data were analyzed using FlowJo software version 10 (BD Biosciences).

Protein extraction and western immunoblot

Erythrocytes from both Duffy-positive (DP) and Duffy-negative (DN) donors were resuspended in lysis buffer and incubated at 4°C for 45 min.[51] Samples were then centrifuged at 1500rpm for 5 min. The supernatants containing the total protein fractions were transferred to new tubes. During differentiation, DP and DN erythroblasts were harvested, and cytoplasmic extracts were obtained using the NE-PER Plus membrane protein extraction kit (Thermo Fisher Scientific) following the manufacturer's instructions. Proteins were quantified using a BCA protein quantification kit (Thermo Fisher Scientific) according to the manufacturer's instructions. For each sample, 10μg of proteins was migrated on a 4-12% SDS-polyacrylamide gel and electroblotted onto a nitrocellulose membrane using the Trans-Blot Turbo system (Bio-Rad). Membranes were blocked in TBS (1X) + 5% milk at room temperature for 60min then probed with rabbit monoclonal anti-DARC antibody (Sigma) in TBS (1X) + 5% milk or mouse monoclonal anti-actin antibody (Cell signaling) in TBS (1X) + 5% BSA at 4°C overnight. Membranes were washed then probed with a goat anti-rabbit antibody (DARC) or goat anti-

CellPress
OPEN ACCESS

mouse antibody (actine, Cell Signaling) at room temperature for 60min. Proteins were detected and visualized using the chemiluminescent detection assay Clarity Max Western ECL (Bio-Rad) and the Chemidoc imaging system (Bio-Rad). Protein quantification was performed using the Image Lab software version 6.1 (Biorad).

Fluorescence microscopy

RNA FISH and antibody staining assay

Cells were harvested and stained to detect the surface expression of DARC, CD71, and *P. vivax*-infected erythroblasts using fluorescence microscopy (RNA Stellaris FISH probes specific to *P. vivax* 18S RNA and anti-Plasmodium HSP70 mouse polyclonal antibody). Briefly, 3×10^6 cells were harvested after 24h and 48h of co-culture and washed with PBS (1X) + 0.2% BSA. The cells were fixed in PBS (1X) supplemented with 4% paraformaldehyde and 0.0075% glutaraldehyde at room temperature for 30 min. The cells were then blocked in PBS (1X) + 3% BSA at room temperature for 60 min. They were then incubated with anti-DARC rabbit monoclonal antibody (Thermo Fisher Scientific) and/or anti-CD71 mouse monoclonal antibody (Thermo Fisher Scientific) at 37°C for 60 min in the dark. The cells were washed twice in PBS (1X) + 1% BSA and incubated with Alexa Fluor 555-conjugated anti-rabbit IgG goat monoclonal antibody and Alexa Fluor 488-conjugated anti-mouse IgG goat monoclonal antibody (Invitrogen) at room temperature for 60 min in the dark. The cells were washed twice in PBS (1X) + 1% BSA and permeabilized in PBS (1X) supplemented with 0.1% Triton (X-100) at room temperature for 15 min. Cells were incubated with HSP70 mouse polyclonal antibody (gift from Olivier Silvie, Biology and Immunology of Malaria Research Unit, CIMI) at 37°C for 60 min in the dark, and then washed twice in PBS (1X). Cells were incubated with Alexa Fluor 680-conjugated anti-mouse IgG goat monoclonal antibody (Invitrogen) at room temperature for 60 min in the dark and then washed twice in PBS (1X). The cells were then hybridized using a set of RNA Stellaris FISH probes specific to *P. vivax* 18S RNA and conjugated to quasar-670 (Stellaris) at 37°C overnight. Cells were washed twice in wash buffer A, incubated at 37°C for 30 min in the dark, and then washed once in wash buffer B. Cells were spun on glass slides at 1000rpm for 3 min using a Cytospin centrifuge (Shandon) and left to dry. They were counterstained using Prolong Gold antifade mountant with diamidino phenylindole (DAPI) (Thermo Fisher Scientific) and covered with coverslips (Epredia). Fluorescence images were captured with a computer-assisted Zeiss Axio Imager.M2 upright microscope equipped with a Plan-Apochromat 63x/1.4 NA Oil objective lens (Carl Zeiss). DAPI images (blue) were collected with a 365 nm excitation filter and a 445/50 nm emmission filter HE Green Fluorescent Prot images (green) with a 475/20 nm excitation filter and a 530/25 nm emmission filter, HE Ds Red images (orange) with a 545/25 nm excitation filter and a 605/70 nm emmission filter), Cy5 images (red) with a 643/30 nm excitation filter and a 690/50 nm emmission filter. The exposure times per channel were kept constant for all the samples to ensure that the intensities could be compared with each other. Brighfield images were collected at exposure time 24.4 ms. Images were captured with a Hamamatsu Camera Orca Flash 4.0lt, controlled, and analyzed using Zen blue software v. 3.7 (Zeiss). A pre-processing pipeline was applied to all 2D images to remove noise, increase contrast, and adjust the dynamic range of the image intensities. For quantitative imaging, we considered the intensity differences when the MFI (Mean Fluorescence Intensity = MFI of the region of interest – MFI of background) was at a minimum x1.5 fold higher with antibody and probe staining.

Statistical analysis

Statistical analyses were performed using GraphPad Prism (GraphPad Software Version 9). Categorical variables were described using frequencies, percentages, and 95% confidence intervals. Chi-square or Fisher's exact tests were used to assess differences in proportion. Continuous variables were summarized as means and standard deviations. The Relative Standard Deviation (RSD) was used to measure the deviation of the proportions disseminated around the mean. For comparisons between two groups, the paired two-tailed t-test or Mann-Whitney test was used. One-way ANOVA variance was performed to compare the three groups. Analysis of covariance (ANCOVA) was used to evaluate changes in the proportion of erythroid cell stages during terminal erythroid differentiation. Statistical significance was considered when the p-value was <0.05.

Cell Host & Microbe, Volume *31*

Supplemental information

Unveiling *P. vivax* invasion pathways in Duffy-negative individuals

Isabelle Bouyssou, Sara El Hoss, Cécile Doderer-Lang, Matthieu Schoenhals, Lova Tsikiniaina Rasoloharimanana, Inès Vigan-Womas, Arsène Ratsimbasoa, Andargie Abate, Lemu Golassa, Solenne Mabilotte, Pascal Kessler, Micheline Guillotte-Blisnick, Francisco J. Martinez, Chetan E. Chitnis, John Strouboulis, and Didier Ménard

Supplemental information

Unveiling *P. vivax* invasion pathways in Duffy-negative individuals

Isabelle Bouyssou, Sara El Hoss, Cécile Doderer-Lang, Matthieu Schoenhals, Lova Tsikiniaina Rasoloharimanana, Inès Vigan-Womas, Arsène Ratsimbasoa, Andargie Abate, Lemu Golassa, Solenne Mabilotte, Pascal Kessler, Micheline Guillotte-Blisnick, Francisco J. Martinez, Chetan E. Chitnis, John Strouboulis and Didier Ménard

Supplemental materials

Table S1 (Related to Figure 4): List of *P. vivax* isolates and erythroblasts used for *in vitro* invasion assays.

***P. vivax* isolate**	**Origin**	**Invasion Assay**	**HSC source**	***P. vivax* infection detection**					**Shown in**	**Comment**
				**MGG staining**	**18S rRNA FISH probe**	**anti-HSP70**	**18S rRNA FISH probe & anti-HSP70**	**Successful**		
AW007	Ethiopia	No. 6	DP010	Negative	**Positive**	Negative	Not done	**Yes**		
			DN005	Negative	Negative	Negative	Not done	No		
MC4023	Ethiopia	No. 5	DP007	**Positive**	**Positive**	Negative	Not done	**Yes**	Figure S7B	Tiny pellet obtained after thawing of the cryoisolate
			DN006	Negative	Negative	Negative	Not done	No		
MC4038	Ethiopia	No. 6	DP010	**Positive**	**Positive**	**Positive**	**Positive**	**Yes**	Figure 4B	
			DN005	**Positive**	**Positive**	**Positive**	**Positive**	**Yes**	Figure 4C	
MC4043	Ethiopia	No. 5	DP007	Negative	Negative	Negative	Not done	No		Lots of red blood cell aggregates
			DN006	Negative	Negative	Negative	Not done	No		
MC4049	Ethiopia	No. 5	DP007	**Positive**	Negative	Negative	Not done	**Yes**		
			DN006	Negative	Negative	Negative	Not done	No		
WN5042	Ethiopia	No. 6	DP010	**Positive**	**Positive**	Negative	Negative	**Yes**	Figure S6C	
			DN005	**Positive**	**Positive**	Negative	Negative	**Yes**	Figure S7A	
WN5050	Ethiopia	No. 5	DP007	Negative	Negative	Negative	Not done	No		Lots of red blood cell aggregates
			DN006	**Positive**	Negative	**Positive**	Not done	**Yes**	Figure S7C	
MDZV02004	Madagascar	No. 5	DP007	Negative	Not done	Not done	Not done	No		Tiny pellet obtained after thawing of the cryoisolate
			DN006	Negative	Negative	Negative	Not done	No		
		No. 6	DP010	Negative	Negative	**Positive**	Not done	**Yes**	Figure S6A	
			DN005	Negative	Negative	**Positive**	Not done	**Yes**	Figure S6B	
MDZV02005	Madagascar	No. 5	DP007	**Positive**	**Positive**	**Positive**	**Positive**	**Yes**	Figure 4E	
			DN006	**Positive**	Negative	**Positive**	Negative	**Yes**	Figure 4G	
		No. 6	DP010	**Positive**	**Positive**	Negative	Negative	**Yes**	Figure 4D	
			DN005	**Positive**	**Positive**	**Positive**	**Positive**	**Yes**	Figure 4F	

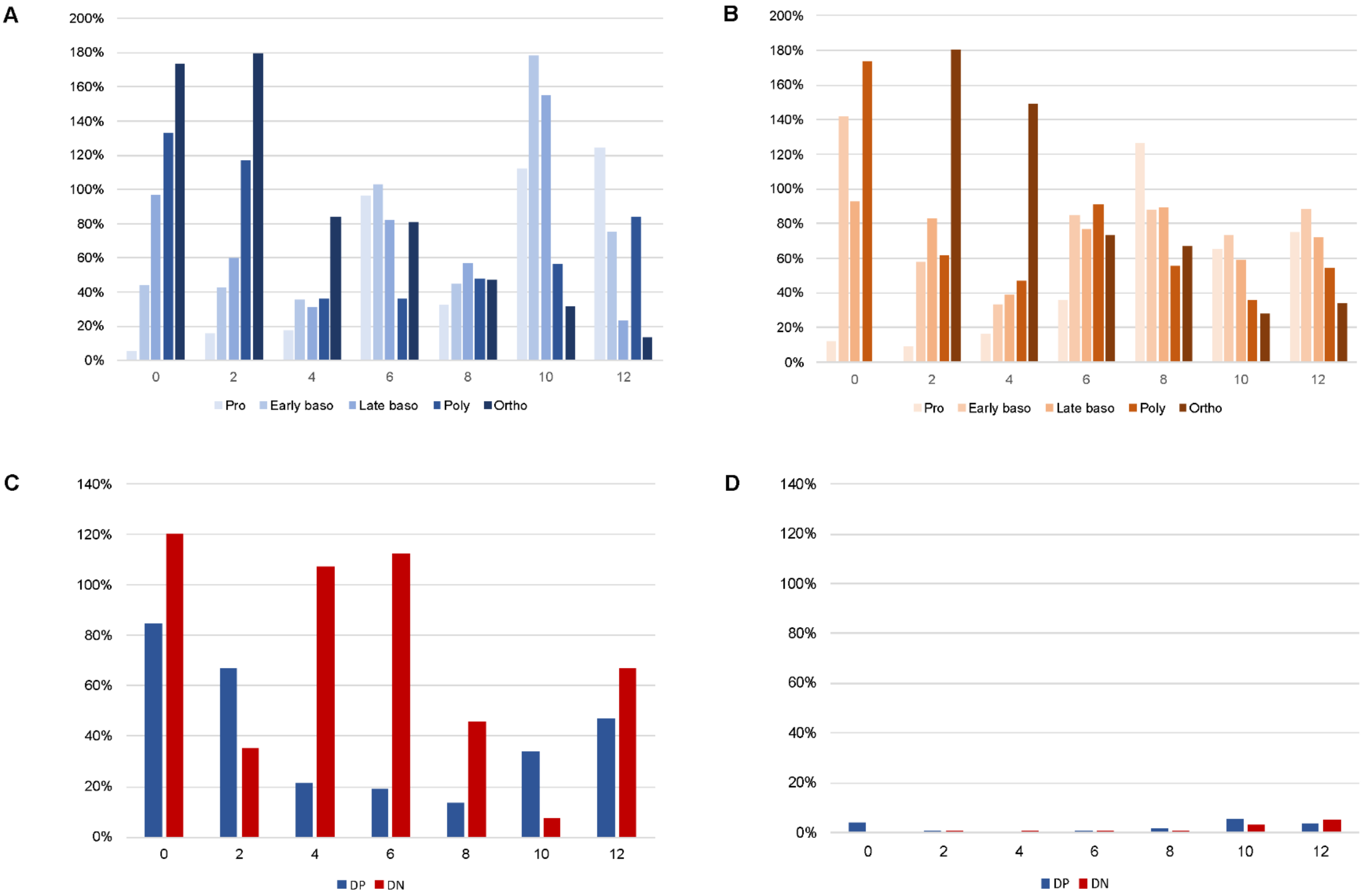
A
200%
180%
160%
140%
120%
100%
80%
60%
40%
20%
0%
0
2
4
6
8
10
12
Pro
Early baso
Late baso
Poly
Ortho
B
200%
180%
160%
140%
120%
100%
80%
60%
40%
20%
0%
0
2
4
6
8
10
12
Pro
Early baso
Late baso
Poly
Ortho
C
140%
120%
100%
80%
60%
40%
20%
0%
0
2
4
6
8
10
12
DP
DN
D
140%
120%
100%
80%
60%
40%
20%
0%
0
2
4
6
8
10
12
DP
DN

Figure S1 (Related to Figure 1 and Figure 2): Inter-individual variation within Duffy-positive (DP) and Duffy-negative (DN) erythroblasts was expressed as relative standard deviations [RSD, RSD=100 × (SD/mean)]. A. RSD of the proportions of the DP erythroblast stages from D0 to D12. **B.** RSD of the proportions of the DN erythroblast stages from D0 to D12. **C.** RSD of the proportion of DP and DN DARC+ erythroblast stages from D0 to D12. **D.** RSD of the proportion of the DP and DN CD71+ erythroblast stages from D0 to D12.

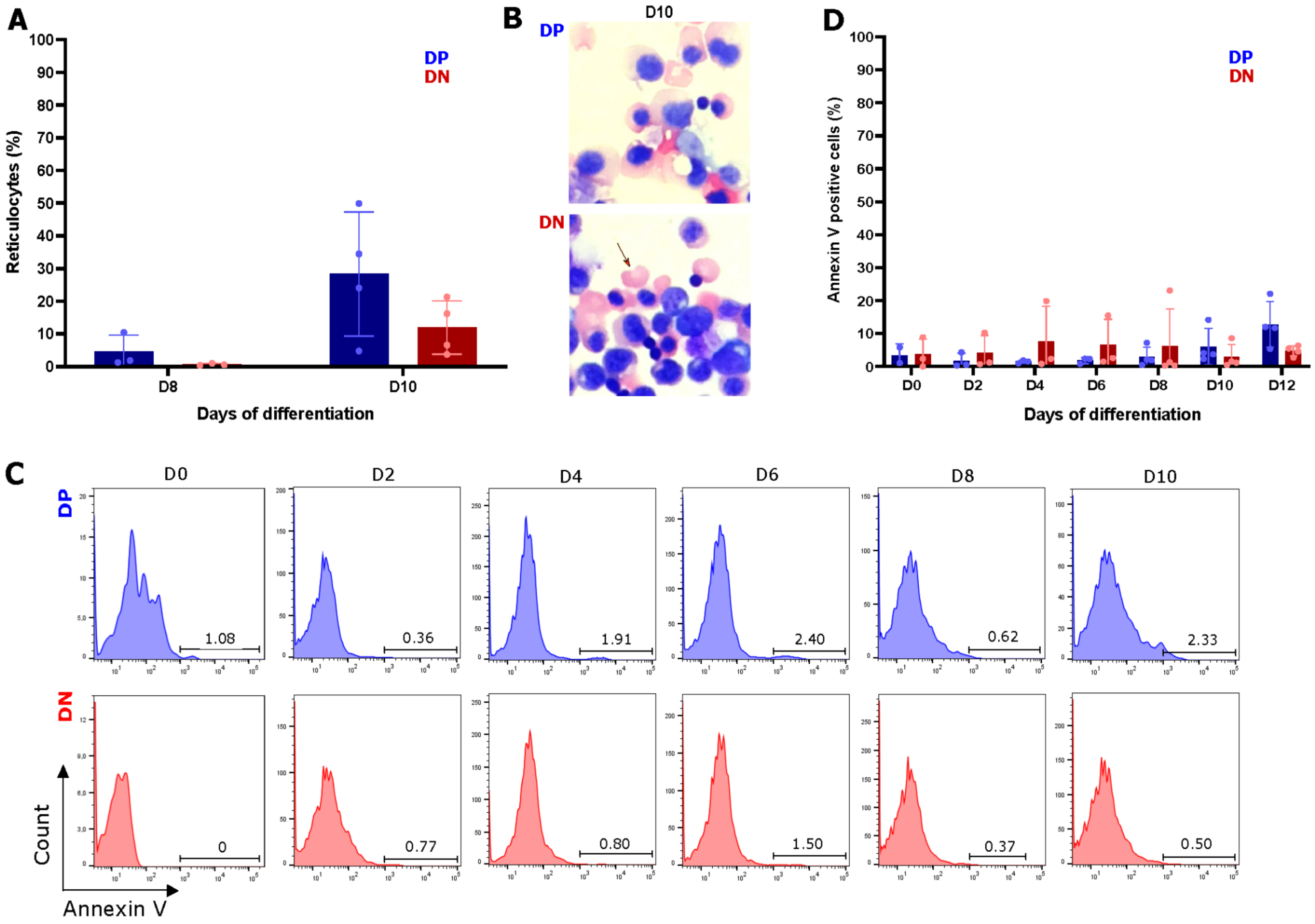

A
Reticulocytes (%)
DP
DN
D8
D10
Days of differentiation
B
D10
DP
DN
D
Annexin V positive cells (%)
DP
DN
D0
D2
D4
D6
D8
D10
D12
Days of differentiation
C
D0
D2
D4
D6
D8
D10
DP
1.08
0.36
1.91
2.40
0.62
2.33
DN
0
0.77
0.80
1.50
0.37
0.50
Count
Annexin V

Figure S2 (Related to Figure 1): Enucleation and apoptosis of Duffy-positive (DP) and Duffy-negative (DN) erythroblasts during terminal differentiation *in vitro*. **A.** The bar chart represents the percentage of reticulocytes in four DP (*DP001, DP002, DP003, DP004*) and four DN (*DN001, DN002, DN003, DN005*) donors at D8 and D10 (mean ±SD) (p=0.2, Mann-Whitney test). **B.** May-Grünwald Giemsa staining showing one DP (*DP001*) and one DN (*DN001*) donors at D10 of terminal differentiation. Arrow indicates the presence of reticulocytes. **C.** Representative histograms showing the percentage of GPA+ Annexin + cells (apoptotic cells) in one DP (*DP003*) and one DN (*DN003*) donor at D0, D2, D4, D6, D8, D10, and D12. **D.** The bar graph represents the percentage of GPA+ Annexin + cells (apoptotic cells) in four DP (*DP001, DP002, DP003, DP004*) and four DN (*DN001, DN002, DN003, DN005*) donors at D0, D2, D4, D6, D8, D10 and D12 (mean ±SD).

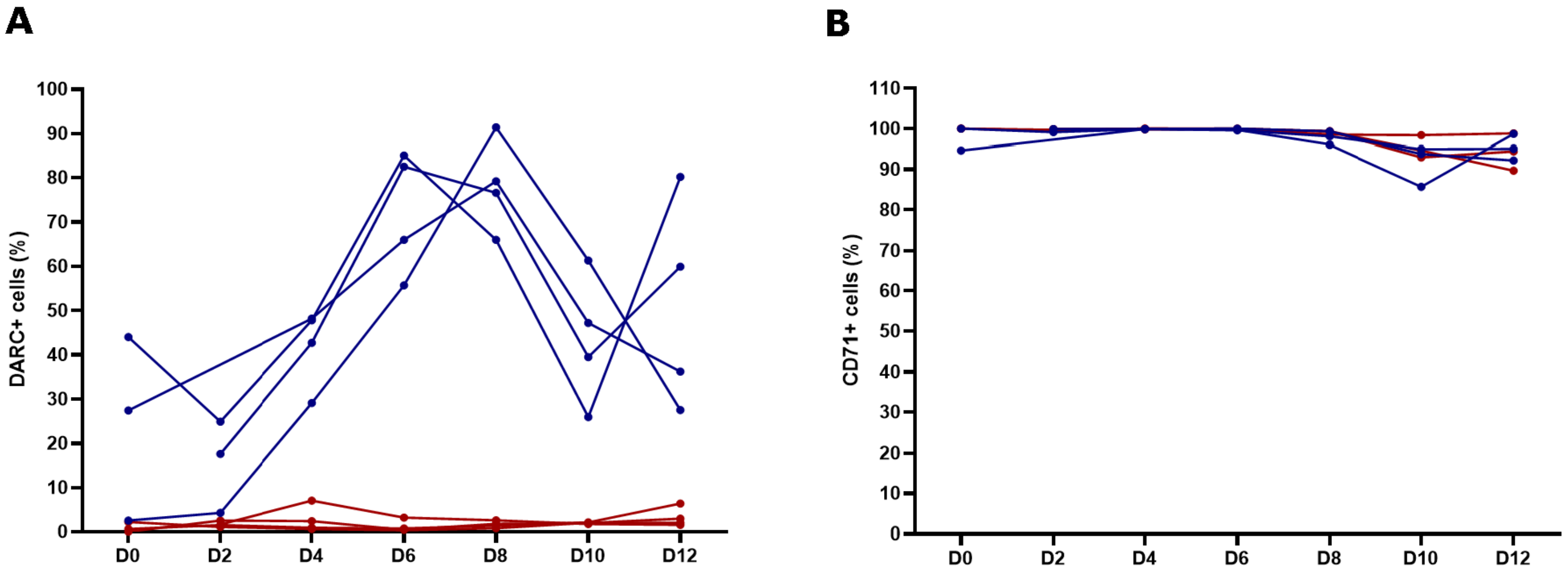

Figure S3 (Related to Figure 2): Expression of DARC and CD71 in Duffy-positive (DP) and Duffy-negative (DN) erythroblasts during terminal differentiation. A. The graphs represent the percentage of GPA+ DARC+ cells in four DP (*DP001, DP002, DP003, DP004*, blue lines) and four DN (*DN001, DN002, DN003, DN005*, red lines) donors at D0, D2, D4, D6, D8, D10 and D12. **B.** The graphs represent the percentage of GPA+ CD71+ cells in three DP (*DP002, DP003, DP004*, blue lines) and three DN (*DN002, DN003, DN005*, red lines) donors at D0, D2, D4, D6, D8, D10 and D12.

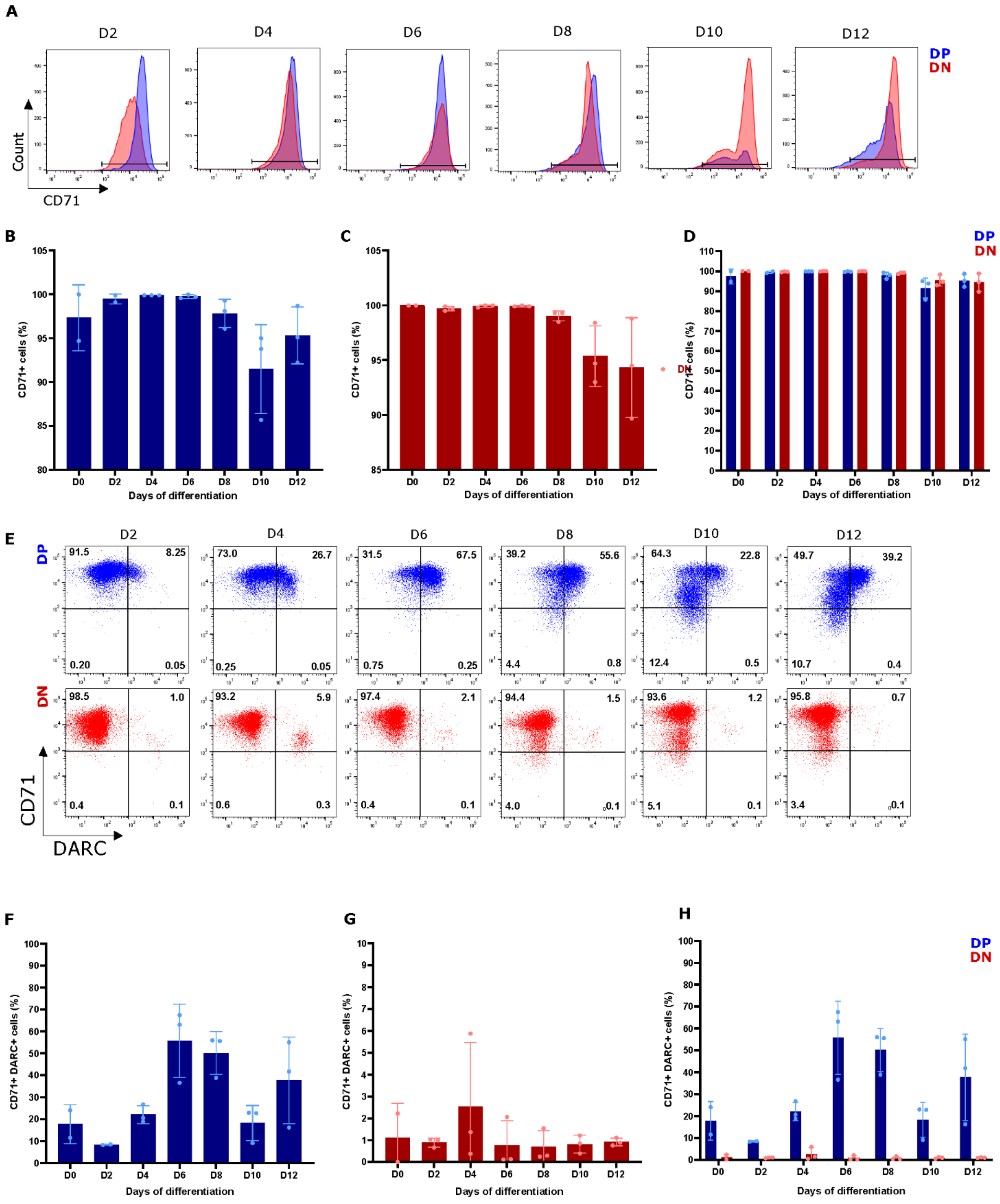

Figure S4 (Related to Figure 2 and Figure 3): Correlation between the expression of DARC and CD71 by Duffy-positive (DP) and Duffy-negative (DN) erythroblasts during terminal differentiation. A. The representative histograms show the percentage of GPA+ CD71+ cells in one DP (*DP004*) and one DN (*DN005*) donors at D0, D2, D4, D6, D8, D10 and D12. **B-D.** The bar charts represent the percentage of GPA+ CD71+ cells in three DP (*DP002, DP003, DP004*, blue bars)

and three DN (*DN002, DN003, DN005*, red bars) donors at D0, D2, D4, D6, D8, D10, and D12 (mean ± SD). **E.** A representative graph showing the distribution of GPA+ cells in one DP (*DP004*) and one DN (*DN005*) donors with respect to the expression of DARC (x-axis) and CD71 (y-axis) at D2, D4, D6, D8, D10 and D12**. F-H.** The bar charts represent the percentage of GPA+ CD71+ DARC+ cells in three DP (*DP002, DP003, DP004,* blue bars) and three DN (*DN002, DN003, DN005*, red bars) donors at D2, D4, D6, D8, D10, and D12 (mean ±SD).

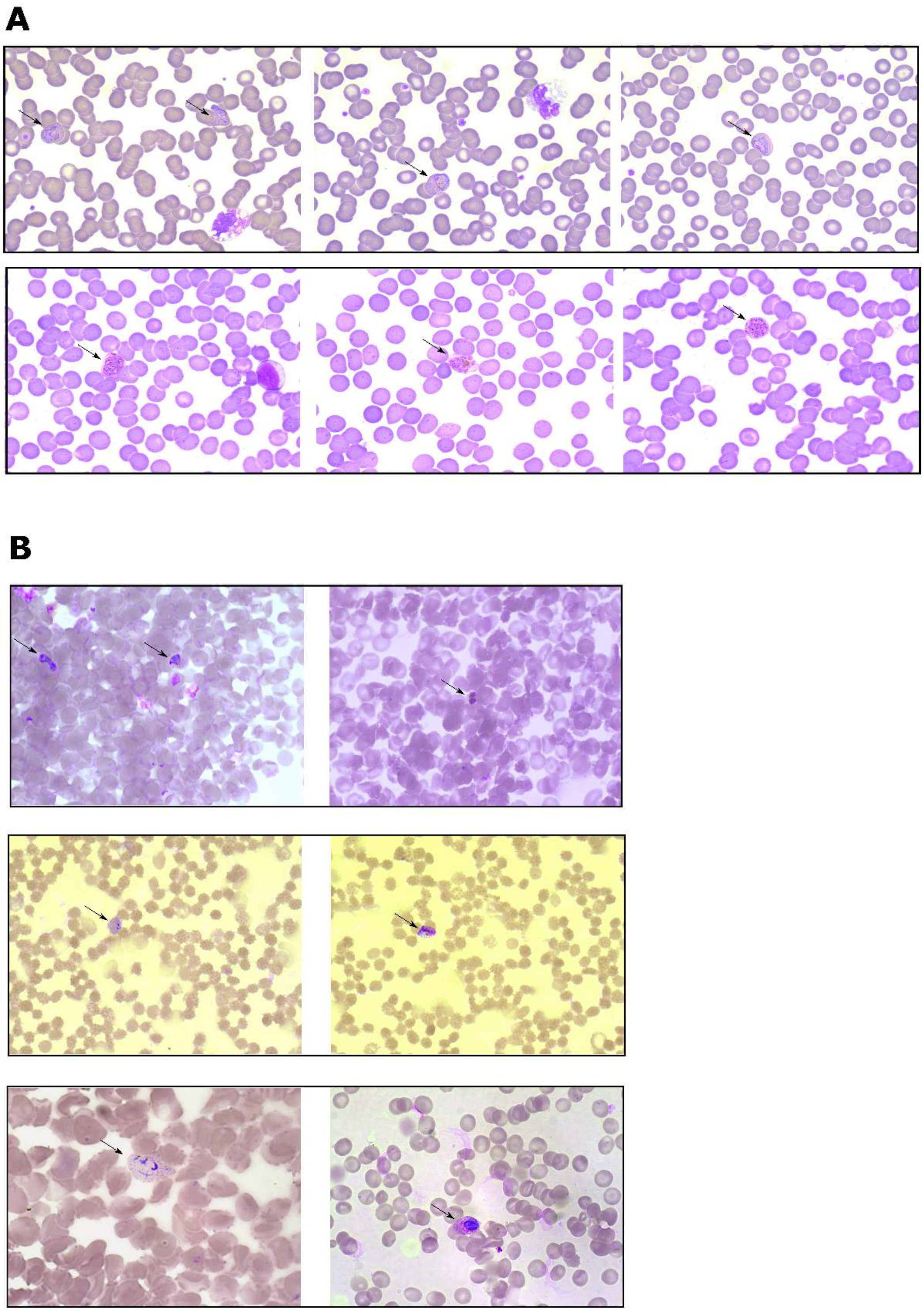

Figure S5 (Related to Figure 4): *P. vivax* parasites from Malagasy and Ethiopian cryoisolate blood samples after thawing and maturation (light microscopy). A. May-Grünwald Giemsa staining showing two Malagasy *P. vivax* isolates, respectively *MDZV02005* (first-line panel) and *MDZV02004* (second-line panel), after thawing. **B.** May-Grünwald Giemsa staining showing one Ethiopian *P. vivax* isolate (*MC4038*, first-line panel) and two Malagasy *P. vivax* isolates (*MDZV02004* and *MDZV02005*, second and third- line panel), after thawing and *in vitro* maturation for 24-30h.

A - coculture DP010 - MDZV02004

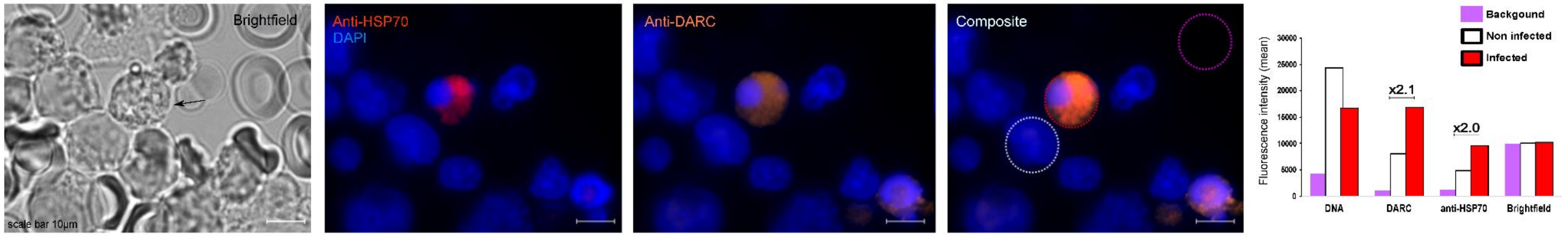

B - coculture DN005 - MDZV02004

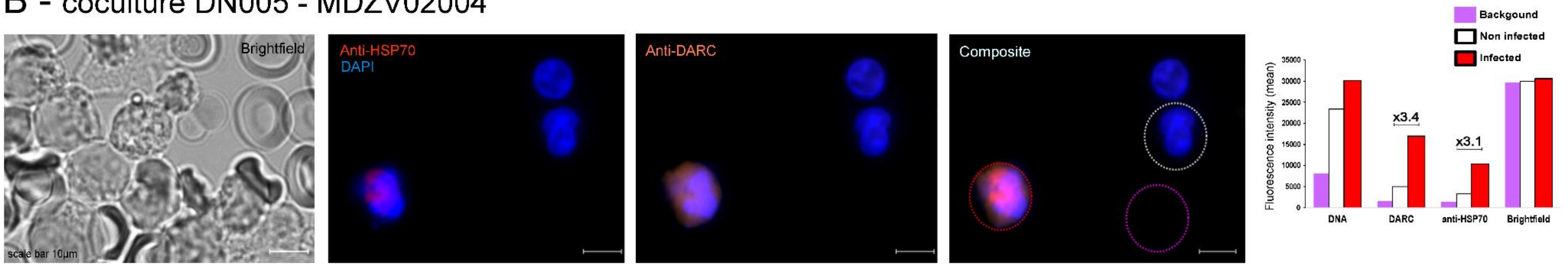

C - coculture DP010 - WN5042

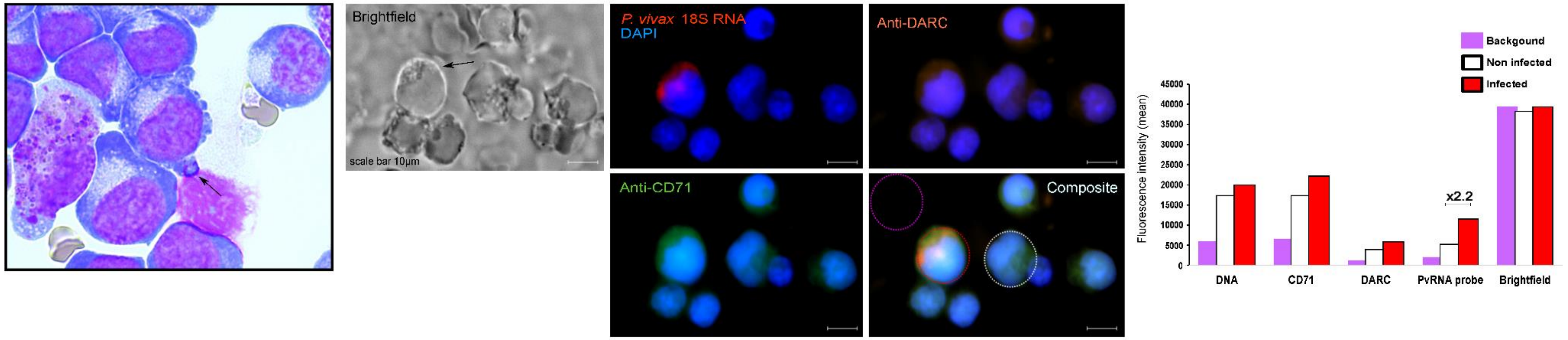

Figure S6 (Related to Figure 4): Additional *P. vivax* infections of Duffy-positive (DP) and Duffy-negative (DN) erythroblasts with Malagasy and Ethiopian *P. vivax* isolates (fluorescence microscopy). A to C. After 24h of co-culture, *P. vivax* parasites were observed on May Grunwald-Giemsa-stained slides by light microscopy and fluorescence microscopy [Brighfield + DAPI (blue)/RNA 18S FISH probe (red) + anti-DARC antibody (orange) + anti-CD71 antibody (green) or Brighfield + DAPI (blue)/anti-HSP70 antibody (red) + anti-DARC antibody (orange), objective 63X]. The bar graphs represent the mean fluorescence intensity (MFI) of the background (purple), non-infected cells (white), and P. vivax-infected cells (red) detected by fluorescence microscopy [DAPI (DNA, blue), RNA 18S FISH probe (red), anti-HSP70 antibody (red or green), anti-DARC antibody (orange), and anti-CD71 antibody (green)]. A scale bar is shown on each image. Additional details are provided in the Method details section and Table S1. **A.** *Co-culture DP010 - MDZV02004*. DP erythroblasts (at D9 of terminal differentiation) infected with a Malagasy *P. vivax* isolate. **B.** *Co-culture DN005 - MDZV02004*. DN erythroblasts (at D9 of terminal differentiation) infected with the same Malagasy *P. vivax* isolate. **C.** *Co-culture DP010 – WN5042*. DP erythroblasts (at D9 of terminal differentiation) were infected with Ethiopian *P. vivax* isolate and observed using light microscopy (mature ring stage) and fluorescence microscopy.

A - coculture DN005 - WN5042

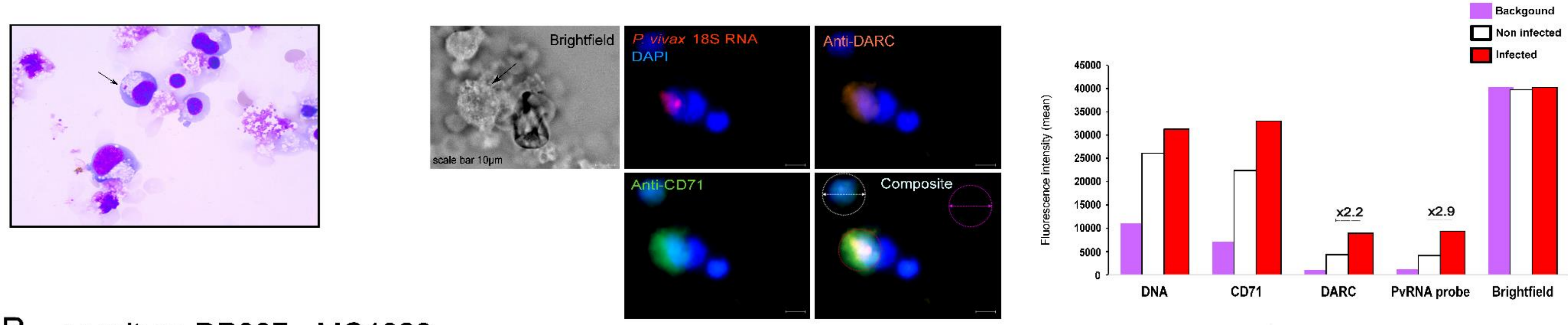

B - coculture DP007 - MC4023

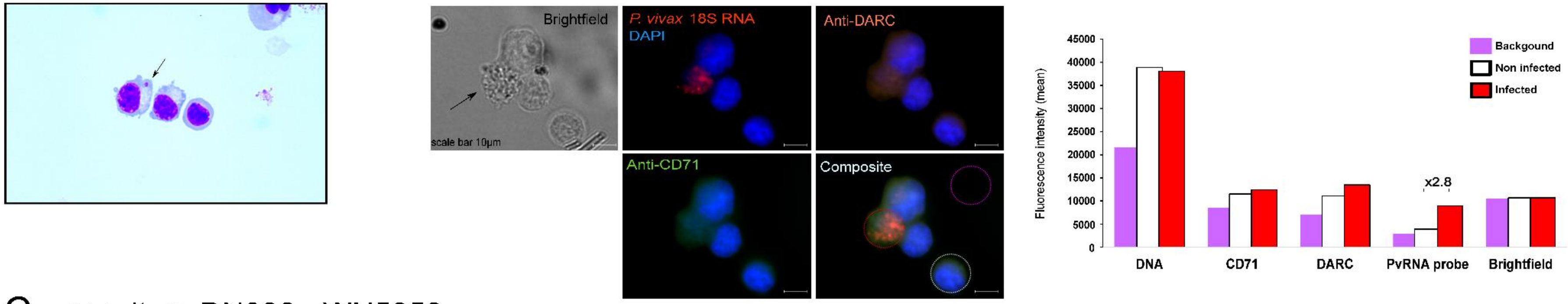

C - coculture DN006 - WN5050

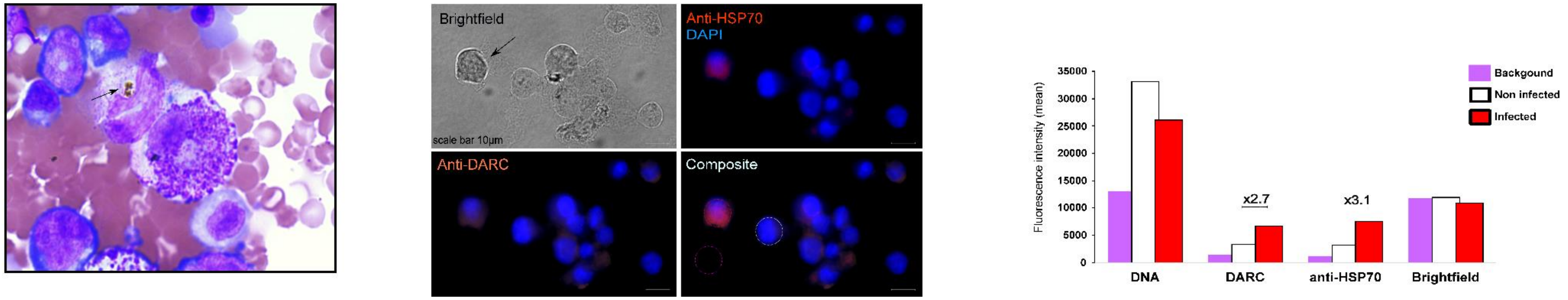

Figure S7 (Related to Figure 4): Additional *P. vivax* infections of DP and DN erythroblasts with the Ethiopian *P. vivax* isolates (light and fluorescence microscopy). A to C. After 48h of co-culture, *P. vivax* parasites were observed on May Grunwald-Giemsa-stained slides by light microscopy and fluorescence microscopy [Brightfield + DAPI (blue)/RNA 18S FISH probe (red) + anti-DARC antibody (orange) + anti-CD71 antibody (green) or Brightfield + DAPI (blue)/anti-HSP70 antibody (red) + anti-DARC antibody (orange), objective 63X]. The bar graphs represent the mean fluorescence intensity (MFI) of the background (purple), non-infected cells (white), and *P. vivax-i*nfected cells (red) detected by fluorescence microscopy [DAPI (DNA, blue), RNA 18S FISH probe (red), anti-HSP70 antibody (red or green), anti-DARC antibody (orange), and anti-CD71 antibody (green)]. A scale bar is shown on each image. Additional details are provided in the Method details section and Table S1. **A.** *Co-culture DN005 – WN5042*. DN erythroblasts (at D9 of terminal differentiation) were infected with Ethiopian *P. vivax* isolate by light microscopy (ring stage) and fluorescence microscopy. **B.** *Co-culture DP007 – MC4023.* DP erythroblasts (at D9 of terminal differentiation) were infected with Ethiopian *P. vivax* isolate and observed using light microscopy (mature stage with hemozoin) and fluorescence microscopy. **C.** *Co-culture DN006 – WN5050*. DN erythroblasts (at D9 of terminal differentiation) were infected with Ethiopian *P. vivax* isolate and observed by light microscopy (mature stage with hemozoin) and fluorescence microscopy.
